# Zipper head mechanism of telomere synthesis by human telomerase

**DOI:** 10.1038/s41422-021-00586-7

**Published:** 2021-11-15

**Authors:** Futang Wan, Yongbo Ding, Yuebin Zhang, Zhenfang Wu, Shaobai Li, Lin Yang, Xiangyu Yan, Pengfei Lan, Guohui Li, Jian Wu, Ming Lei

**Affiliations:** 1 Ninth People’s Hospital, Shanghai Jiao Tong University School of Medicine, Shanghai, China; 2 Shanghai Institute of Precision Medicine, Shanghai, China; 3 CAS Center for Excellence in Molecular Cell Science, Shanghai Institute of Biochemistry and Cell Biology, Chinese Academy of Sciences, Shanghai, China; 4 University of Chinese Academy of Sciences, Beijing, China; 5 Laboratory of Molecular Modeling and Design, State Key Laboratory of Molecular Reaction Dynamics, Dalian Institute of Chemical Physics, Chinese Academy of Sciences, Dalian, Liaoning China; 6 Renji Hospital, Shanghai Jiao Tong University School of Medicine, Shanghai, China; 7 State Key Laboratory of Oncogenes and Related Genes, Shanghai Jiao Tong University School of Medicine, Shanghai, China

**Keywords:** Cryoelectron microscopy, Telomeres

## Abstract

Telomerase, a multi-subunit ribonucleoprotein complex, is a unique reverse transcriptase that catalyzes the processive addition of a repeat sequence to extend the telomere end using a short fragment of its own RNA component as the template. Despite recent structural characterizations of human and *Tetrahymena* telomerase, it is still a mystery how telomerase repeatedly uses its RNA template to synthesize telomeric DNA. Here, we report the cryo-EM structure of human telomerase holoenzyme bound with telomeric DNA at resolutions of 3.5 Å and 3.9 Å for the catalytic core and biogenesis module, respectively. The structure reveals that a leucine residue Leu980 in telomerase reverse transcriptase (TERT) catalytic subunit functions as a zipper head to limit the length of the short primer–template duplex in the active center. Moreover, our structural and computational analyses suggest that TERT and telomerase RNA (hTR) are organized to harbor a preformed active site that can accommodate short primer–template duplex substrates for catalysis. Furthermore, our findings unveil a double-fingers architecture in TERT that ensures nucleotide addition processivity of human telomerase. We propose that the zipper head Leu980 is a structural determinant for the sequence-based pausing signal of DNA synthesis that coincides with the RNA element-based physical template boundary. Functional analyses unveil that the non-glycine zipper head plays an essential role in both telomerase repeat addition processivity and telomere length homeostasis. In addition, we also demonstrate that this zipper head mechanism is conserved in all eukaryotic telomerases. Together, our study provides an integrated model for telomerase-mediated telomere synthesis.

## Introduction

Eukaryotic linear chromosomes face two major challenges, known as the end-replication and end-protection problems.^1,2^ Telomeres, highly ordered DNA–protein complexes located at chromosomal ends, are evolved to solve these two problems and ensure complete genome replication and genome stability.^3^ Telomeric DNAs consist of a track of double-stranded G-rich repeats, and terminate in a 3′ single-stranded (ss) overhang.^4^ In most eukaryotes, telomeric DNAs are synthesized by telomerase, a multi-subunit ribonucleoprotein (RNP) complex with a reverse transcriptase activity using its own RNA component as the template.^5–7^ Human telomerase holoenzyme consists of the telomerase reverse transcriptase (TERT) catalytic subunit, the telomerase RNA (hTR) component, two sets of H/ACA proteins (dyskerin, GAR1, NHP2 and NOP10), and telomerase Cajal body protein 1 (TCAB1).^7–10^ Telomerase is activated in continually dividing cells, such as human stem cells and germ cells, to counteract telomere loss during replication.^11^ Telomerase deficiency is linked to several human telomere syndromes including dyskeratosis congenita and Hoyeraal-Hreidarsson syndrome.^12,13^ In contrast, telomerase activity is repressed in somatic cells to prevent uncontrolled telomere lengthening that would lead to cell immortalization.^14^ Telomerase activity in somatic cells is tumorigenic and upregulated telomerase activity is observed in > 80% cancers.^15^ Therefore, telomerase has been considered as a potential universal therapeutic target for cancers.

Distinct from canonical protein-only reverse transcriptases (RTs), a salient feature of telomerase is that, in addition to nucleotide addition processivity (NAP), it catalyzes the processive addition of a repeat sequence to extend the telomeric overhang, a process referred to as repeat addition processivity (RAP).^16^ In this unique process, the RNA template fragment is recycled after each telomeric repeat addition by translocating back to its initial position to realign with the telomere end for next round of repeat synthesis.^17,18^ The RNA subunit of telomerase contains a 5′ template-flanking sequence known as the template boundary element (TBE) that defines the physical template boundary at the end of each repeat synthesis.^19–21^ Strikingly, recent biochemical studies have revealed a sequence-derived pausing signal that define the same template boundary as TBE.^22,23^ However, the mechanism and structural determinant of this sequence-based template boundary still remains enigmatic.

It is well known that canonical RTs require a primer–template duplex of at least 6 base pairs to initiate DNA synthesis.^24^ Surprisingly, human telomerase only needs a 3-base-paired primer–template duplex to sustain its catalytic activity.^25^ Moreover, previous biochemical data demonstrated that the base pairing between primer and template makes minimal contribution to the stabilization of the duplex.^25^ Although several lines of evidence demonstrate that the telomerase essential N-terminal (TEN) domain of human TERT plays an important role in stabilizing the primer–template hybrid in the active site,^18,26,27^ it is still not clear how human telomerase utilizes such a short primer–template duplex as the substrate. In fact, the length of the primer–template duplex during telomere synthesis remains unresolved.

Here, we present the cryo-EM structure of human telomerase holoenzyme with bound telomeric DNA at resolutions of 3.54 Å and 3.94 Å for the catalytic core and the biogenesis module, respectively. Our work provides a mechanistic understanding of the catalytic cycle of telomerase-mediated telomere repeat synthesis.

## Results

### Structural determination of human telomerase

For structural characterization of human telomerase, we first generated a HEK293F cell line stably expressing Strep-tagged TCAB1, which is essential for the biogenesis of hTR.^10^ We then reconstituted human telomerase holoenzyme by transient transfection of this TCAB1 cell line with vectors expressing hTR and Flag-tagged TERT in a suspension culture system. Two-step tandem affinity purification allowed us to purify human telomerase RNP for structural studies (Supplementary information, Fig. S1a). The purified holoenzyme was then subjected to mass spectrometry analysis, confirming the presence of all previously identified protein components in human telomerase (Supplementary information, Fig. S1b). The quality of the telomerase RNP was further affirmed by visual inspection of negative staining micrographs, which exhibited homogeneous particles with the expected size (Supplementary information, Fig. S1c).

**Fig. 1:**
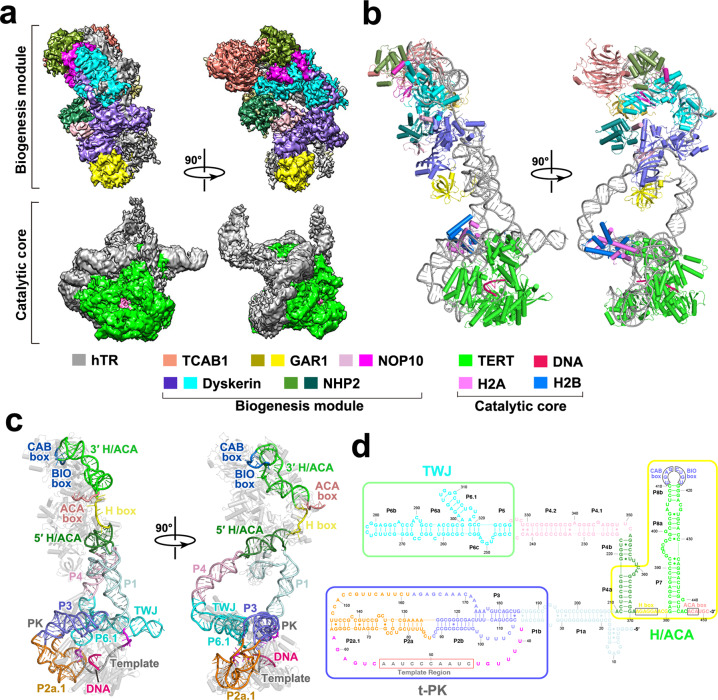
Overall structure of human telomerase holoenzyme in complex with the ssDNA substrate. **a**, **b** EM density map (**a**) and atomic model (**b**) of human telomerase holoenzyme are shown in two orthogonal views. Protein and RNA subunits are color coded, and the scheme is shown below the figure. **c** Two orthogonal views of the overall structure of the hTR RNA. Conserved motifs are highlighted by different colors. **d** Secondary structure diagram of the hTR RNA. RNA elements are colored as in **c**. The t-PK, TWJ and H/ACA domains are denoted. Canonical Watson-Crick and non-canonical base-pairing interactions are shown as solid lines and dots, respectively.

To gain insights into the architecture of human telomerase and the molecular basis of its unique RAP, we set out to determine the cryo-EM structure of human telomerase holoenzyme in complex with a DNA substrate (T_20_AGGG). Our two-dimensional classification analysis of cryo-EM data unveiled a highly dynamic architecture with two lobes, which respectively corresponds to the catalytic core and the biogenesis module of the RNP (Supplementary information, Fig. S1d).^7,8^ Local focused classification of the two lobes enabled us to determine the structures of the catalytic and the biogenesis modules of human telomerase at resolutions of 3.54 Å and 3.94 Å, respectively (Fig. 1a, b; Supplementary information, Figs. S1e, S2–S4 and Table S1). We connected the two domains together to assemble the entire atomic model of human telomerase RNP using modeled stems P1 and P4.1–4.2 (Fig. 1a, b). Consistent with the recent report of human telomerase structure,^29^ during cryo-EM data processing we observed extra densities with histone fold features adjacent to hTR in ~60% of the processed particles (Supplementary information, Figs. S2–S5). Notably, histone H2A–H2B dimer, but not the H3–H4 dimer, could snugly fit into the density, in accordance with the mass spectrometry data that only histones H2A and H2B were identified with high abundance in the purified human telomerase (Supplementary information, Fig. S1b). Together, the final refined atomic model of the human telomerase holoenzyme contains all previously identified components (hTR, TERT, TCAB1 and two sets of H/ACA proteins), a telomeric DNA substrate and the histone H2A–H2B heterodimer (Fig. 1b; Supplementary information, Fig. S1e).

### Overall architecture of human telomerase

The 451-nucleotide (nt) hTR RNA is composed of three structural domains, the template-pseudoknot (t-PK) and three-way junction (TWJ) domains in the catalytic core and the H/ACA domain in the biogenesis module (Fig. 1c, d). The hTR RNA adopts a fully extended conformation with 11 stems, spanning more than 250 Å (Fig. 1c). In addition, there are two long ss regions in the t-PK domain, the template as well as flanking sequences and the fragment between stems P3 and P2a.1; the 5′ ends of both ss fragments are disordered in the EM structure (Fig. 1c, d). The most salient feature of hTR is that there are no contacts amongst the hTR structural motifs, but instead all these elements mediate intimate interactions with their protein partners (Fig. 1a–c). The TERT protein simultaneously contacts the t-PK and the TWJ domains of hTR to form the catalytic core of the holoenzyme with a short primer–template duplex inside the central hole of TERT (Fig. 1a–c). In some human telomerase complexes, the TWJ domain is also stabilized by the H2A–H2B heterodimer (Fig. 1b, c). On the other side of the holoenzyme, two sets of H/ACA proteins (dyskerin, GAR1, NOP10 and NHP2) respectively bind the two H/ACA hairpins of hTR in a tandem fashion, forming the biogenesis module (Fig. 1a–c). The 3′ H/ACA unit has an extra protein component TCAB1 that binds the CAB box at the terminal loop of the RNA hairpin (Fig. 1a–c). Interestingly, the 5′ tail of hTR, previously predicted to potentially form a G-quadruplex,^30,31^ folds into a short stem and is stabilized by an arginine-rich motif of GAR1 in the 5′ H/ACA unit (Supplementary information, Fig. S4). Given that the G-rich 5′ tail and the arginine-rich motif of GAR1 are conserved in most vertebrate telomerases, it is likely that this unique RNP motif could provide a universal protection mechanism for the 5′ end of vertebrate TRs.

### Structure of the biogenesis module

**Fig. 2:**
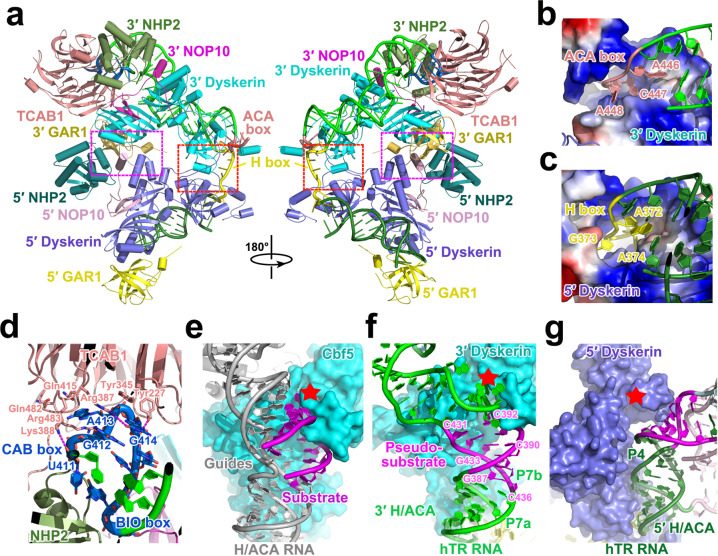
EM structure of the biogenesis module of human telomerase. **a** Front and back views of the biogenesis module with protein factors colored as in Fig. 1. The two protein–protein interfaces between the two H/ACA units are highlighted with dashed magenta and red boxes, respectively. **b**, **c** Close-up views of the ACA (**b**) and H (**c**) boxes that are recognized by 3′ and 5′ dyskerins via sequence-specific interactions, respectively. The two dyskerin proteins are shown in electrostatic surface potentials (positive potential, blue; negative potential, red). **d** Close-up view of the CAB and BIO boxes that are recognized by TCAB1 and NHP2. TCAB1 and NHP2 are shown in ribbon representations, and residues important for the interactions are shown in stick model. **e**–**g** Structural comparison of the 3′- (**f**) and 5′- (**g**) H/ACA units of human telomerase with the canonical substrate-bound H/ACA RNP (**e**). The putative active site for pseudouridine synthases was designated with red star.

In the biogenesis module there are two protein–protein interfaces between the two H/ACA units separated by a big hole in the center (Fig. 2a). One interface is mainly mediated by hydrophilic contacts among protein factors from both units away from the RNA hairpins (Fig. 2a; Supplementary information, Fig. S6a). The other is between the PseudoUridine synthase and Archaeosine transglycosylase (PUA) domains of two dyskerin proteins near the H box of the RNA; the convex helix α10 in the PUA domain from dyskerin that associates with the 3′ hairpin complementarily fits into the concave surface on the opposite side of the PUA domain from the other dyskerin through hydrophobic contacts (Fig. 2a; Supplementary information, Fig. S6b). Consequently, the two H/ACA units are arranged in a tandem manner with a ~55° rotational symmetry (Fig. 2a). This connection between adjacent H/ACA units provides a structural basis for the modular arrangement of eukaryotic H/ACA RNPs with multiple RNA hairpins (Fig. 2a).^32^

In both H/ACA units, the stems of the two RNA hairpins respectively attach on the flat, basic surfaces of two dyskerin proteins through nonspecific electrostatic contacts involving mostly the backbone of the RNA (Supplementary information, Fig. S6c).^33^ In contrast, nucleotides in the H and ACA boxes are recognized by sequence-specific interactions, and partially or completely buried in protein factors (Fig. 2b, c). The ACA box (A446–C447–A448) at the 3′ terminus of hTR snugly nests in a deep cleft in the PUA domain of the 3′ dyskerin similar to the contacts observed in archaeal H/ACA RNP structures (Fig. 2b).^33^ Strikingly, nucleotides A372–G373–A374, corresponding to the 5′ nucleotides in the H box consensus sequence ANANNA, are recognized by the 5′ dyskerin exactly in the same manner as the ACA box by the 3′ dyskerin (Fig. 2b, c). Notably, residues 382–421 C-terminal to the PUA domain in the 5′ dyskerin fold into a small motif (referred to as the H-box-binding motif of dyskerin; HBM), which together with the PUA domain encircle nucleotides A372–G373–A374 (Supplementary information, Fig. S6d). The third conserved nucleotide A377 at the 3′ end of the H box packs against nucleotide C447 to stabilize the ACA box, occupying the equivalent position of Tyr416 in the HBM of 5′ dyskerin (Supplementary information, Fig. S6e).

The terminal loop of hairpin P8b contains the CAB and BIO boxes of hTR, which are required for mature RNA accumulation and binding with the Cajal body localization factor TCAB1, respectively.^10,34^ The CAB and BIO boxes are completely sandwiched between NHP2 and TCAB1 (Fig. 2d). TCAB1 adopts a typical WD40-repeat-domain architecture that contains a seven-bladed β-propeller (Fig. 2a).^35^ The CAB box fits into a narrow cleft on one side of the propeller, with nucleotide A413 pointing into a deep hydrophilic pocket formed by Gln415, Gln482 and Arg483 of TCAB1 (Fig. 2d). Outside of this pocket, the CAB and BIO boxes are mostly stabilized by stacking among RNA bases as well as electrostatic interactions with TCAB1 and NHP2 positively-charged amino acids (Supplementary information, Fig. S6f). The G414C substitution in the CAB box was reported to disrupt the association of TCAB1 with human telomerase, consistent with the observation that G414 mediates a hydrogen-bonding network with the side chain of TCAB1 Arg387.^35^

### Human telomerase is a pseudo-pseudouridine synthase

Compared to canonical H/ACA RNAs, the structures of both 5′ and 3′ H/ACA hairpins in hTR exhibit marked differences in the middle region of the hairpins, which, in canonical H/ACA pseudouridine synthases, forms the pseudouridylation pocket by two separated ss guide RNA fragments with similar lengths (Fig. 2e).^36^ hTR nucleotides G387–C392, which have been predicted as an unpaired fragment, in fact occupy the equivalent positions of the 5′ half of the RNA substrate in canonical H/ACA RNPs and form six consecutive base pairs with C431–C436 from the opposite strand along the helical trajectory of stems P7a–P7b (Fig. 2f). Hence, instead of being a guide RNA, nucleotides C431–C436 in hTR 3′ hairpin function as a pseudo-substrate and block the pseudouridylation pocket of dyskerin (Fig. 2f). This unique pseudo-substrate conformation results in a 5-nt bulge at one side of the 3′ hairpin, forcing the upper P8a–P8b stem to make a sharp turn towards NHP2 and TCAB1 (Fig. 2a, f). Structural comparison reveals that the 5′ H/ACA hairpin also contains a 5-nt bulge in the middle of stem P4, which blocks the pseudouridylation pocket of the 5′ dyskerin as well (Fig. 2g). However, in contrast to the 3′ bulge, this 5′ bulge resides on the other strand of the stem, so that the 5′ H/ACA hairpin makes a turn to the opposite direction towards the catalytic core of the holoenzyme (Fig. 2a, g; Supplementary information, Fig. S6c). Collectively, the atomic structure of the biogenesis module provides a structural explanation why human telomerase is not a pseudouridine synthase.^37^ Sequence and secondary structure analyses suggest that the autoinhibited conformation observed in the biogenesis module of human telomerase is likely a conserved structural feature for all vertebrate TRs (Supplementary information, Fig. S7).

### A built-in zipper head in TERT limits the length of the primer–template duplex

**Fig. 3:**
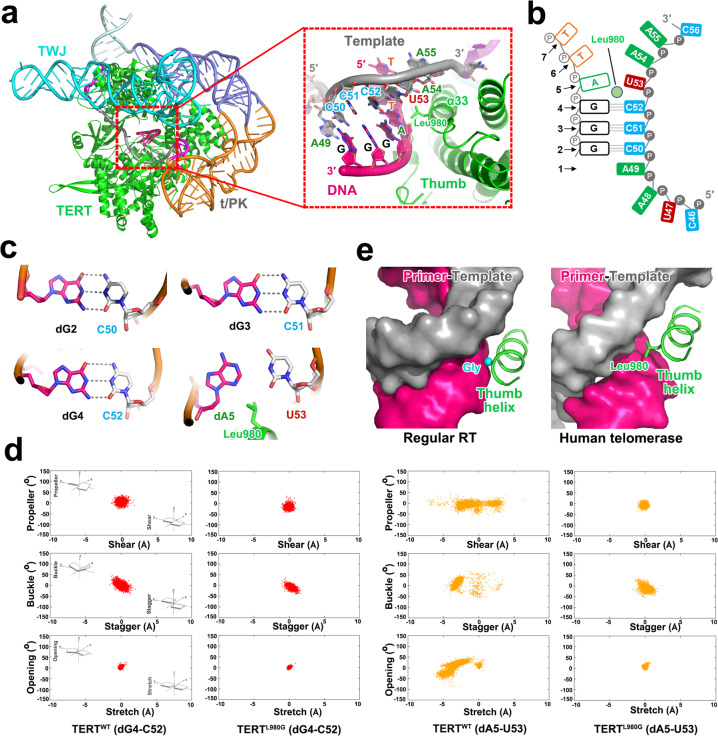
Zipper head residue Leu980 in TERT limits the length of the primer–template duplex. **a** Left, structure of the catalytic core of human telomerase as colored in Fig. 1, with the active cavity of TERT highlighted in dashed red box. Right, close-up view of the primer–template duplex in the active site of TERT. The catalytic center of telomerase is defined as ‘position 1’ which is vacant in the EM structure. The zipper head residue Leu980 in the thumb helix of TERT is shown in stick model. **b** Schematic diagram of the primer–template duplex in the structure in which the zipper head Leu980 (light green circle) destabilizes and disrupts the canonical W-C hydrogen pairing at positions 5 and positions 6 and 7. **c** Details of the base pairing of the primer–template duplex at the AGGG 3′-end of the DNA substrate. The wobble pair dA5–U53 at position 5 no longer mediates canonical W-C hydrogen bonding interactions due to the disruptive effect of Leu980. **d** MD simulation scatter plots of the geometrical base pair descriptors of the RNA–DNA hybrid at positions 4 and 5 in WT and L980G mutant TERT proteins, respectively. **e** Position on the thumb helix equivalent to human TERT^Leu980^ is occupied by an invariant glycine residue (cyan ball) in canonical protein-only RTs.

The cryo-EM density map was of sufficient quality to enable us to build the atomic model of six primer and seven template nucleotides in the active site, unveiling a state during primer extension in which the catalytic center is vacant for the incoming deoxynucleoside triphosphate (dNTP) (Fig. 3a). For simplicity, hereafter we define the catalytic center of telomerase as ‘position 1’ (Fig. 3a, b). Nucleotides dG2–dG3–dG4 in the primer and C50–C51–C52 in the template mediate three consecutive Watson-Crick (W-C) base pairs at positions 2–4 (Fig. 3a–c). Strikingly, the side chain of Leu980 at the C-terminus of helix α33 (thumb helix) in the thumb subdomain of TERT (previously referred to as CTE) sticks deep into the minor groove of the primer–template duplex and pushes nucleotides dA5 and U53 at position 5 towards the major groove so that they no longer mediate canonical W-C hydrogen bonding interactions (Fig. 3a–c). This disruptive effect is beyond position 5; primer and template nucleotides at positions 6 and 7 (dT6, dT7, A54 and A55) are completely separated from each other with no connections in between and therefore make no contribution to the primer–template pairing (Fig. 3a, b). This structural feature is in accordance with previous biochemical data that the stability ranking of the interactions between human telomerase and primers with all six permutations is consistent with the number of hydrogen bonds mediated by the last three nucleotides of the primers.^25^

To corroborate our structural study, we carried out molecular dynamics (MD) simulations to investigate how Leu980 affects the structural stability of the primer–template in the active site. Consistent with the cryo-EM structure, MD simulation trajectories showed that only nucleotides at positions 2–4 were stably maintained in paired conformations, whereas the wobble pair dA5–U53 displayed severe distortions from the canonical W-C conformation during the 500 ns simulation time (Fig. 3d; Supplementary information, Fig. S8). Strikingly, a glycine substitution of Leu980 could effectively restore a 6-bp primer–template duplex in the active site, underscoring the disruptive effect of Leu980 on the substrate conformation (Fig. 3d; Supplementary information, Fig. S8). Taken together, both our cryo-EM structure and MD simulations revealed that TERT^Leu980^ functions as a ‘zipper head’ in the middle of the primer–template duplex so that human telomerase can only accommodate three W-C primer–template base pairs in the active site before dNTP incorporation (Fig. 3a, b). This zipper head mechanism of human telomerase dispels the ‘product–DNA–hairpin’ hypothesis and is more reminiscent of RNA polymerase, which maintains a constant amount of base pairing in a transcription bubble during elongation.^38,39^

The zipper head mechanism suggests that human telomerase is not an efficient polymerase. In the telomere extension reaction, in addition to propelling the translocation of the primer–template duplex that is similar to all other nucleic acid polymerases, the energy available from dNTP incorporation also needs to overcome the resistance of the zipper head residue and destabilizes a base pair during its translocation from position 4 to position 5. Notably, positions on the thumb helix equivalent to human TERT^Leu980^ in other RTs are all occupied by an invariant glycine residue (Fig. 3e).^40^ Even a relatively modest alanine substitution of this glycine resulted in enhanced pausing in DNA synthesis.^40^ Therefore, this key tracking position in the thumb helix is evolved to monitor the minor groove of the nascent duplex to play different roles; in regular RTs a glycine residue serves as an important modulator of both processivity and fidelity, whereas in human telomerase a leucine residue functions as a zipper head to maintain a short primer–template duplex and confers a low NAP. In accordance with this idea, previous studies showed that human telomerase is at least ~20-fold less efficient in NAP than other RTs.^41–43^

### hTR stabilizes TERT for RT activity

**Fig. 4:**
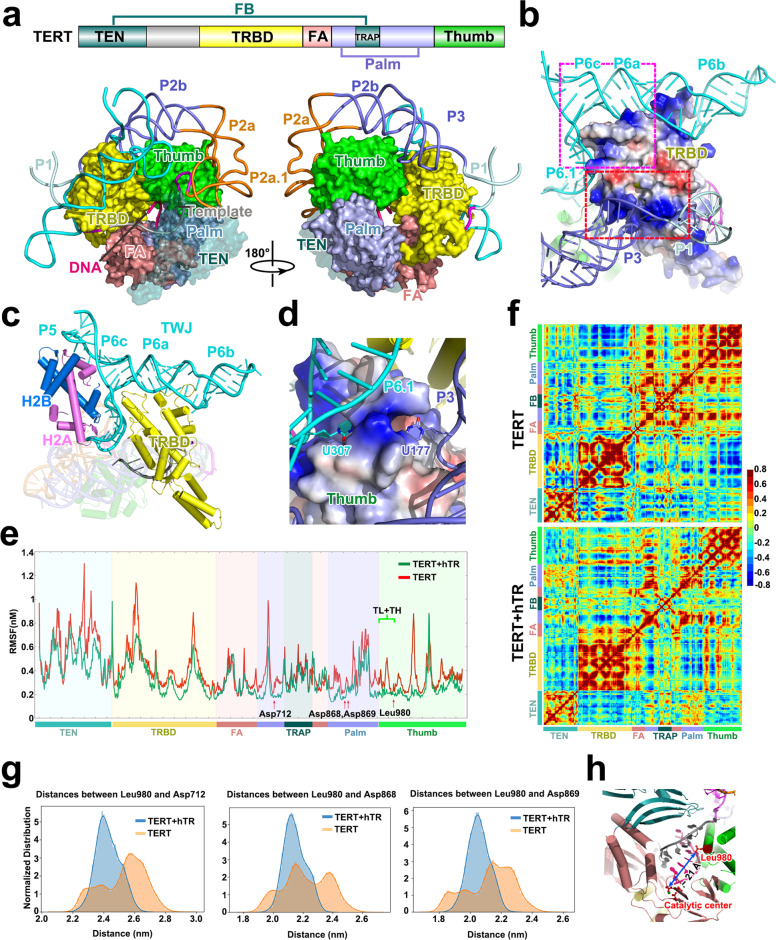
The hTR RNA stabilizes TERT to maintain the short primer–template duplex in the active site. **a** Double-ring architecture of TERT and hTR. Upper, Domain architecture of TERT. TEN, telomerase essential N-terminal domain; TRBD, telomerase RNA-binding domain; FA and FB, fingers-A and fingers-B; Thumb, C-terminal extension (CTE) analogous to polymerase thumb subdomain. Lower, front and back views of the double-ring architecture of TERT and hTR. The domains are colored as in the upper panel. **b** Electrostatic surface potential of hTR-interacting sites of the TRBD subdomain of TERT. The two interacting surfaces of TERT_TRBD_ with the t-PK and TWJ domains of hTR are highlighted with dashed red and magenta boxes, respectively (positive potential, blue; negative potential, red). The hTR RNA is shown in cartoon representation. **c** Interactions of the TWJ domain of hTR with the TERT_TRBD_ and the histone H2A–H2B heterodimer. Proteins are shown in cylinder representation. **d** Close-up view of the interactions between TERT_thumb_ and the t-PK and TWJ domains of hTR. TERT_thumb_ is shown in electrostatic surface potential. **e** Comparison of root-mean-square-fluctuations (RMSFs) of TERT in the presence (green) and absence (red) of hTR. Domain architecture of TERT is shown below the figure. **f** Cross-correlation of TERT in the absence (upper) and presence (lower) of hTR. **g** Distance distributions between the zipper head residue Leu980 and three essential aspartate residues (Asp712, Asp868 and Asp869) at the catalytic center in the absence (orange) and presence (light blue) of hTR. **h** Overall view of the distance between the zipper head Leu980 and the catalytic center.

It is well known that canonical RTs require a primer–template duplex of at least 6 bp to initiate DNA synthesis.^24^ In stark contrast, human telomerase only needs a 3-bp alignment between the primer and the template to sustain its catalytic activity, although a 3-bp DNA–RNA hybrid is energetically barely stable to maintain a duplex conformation (Fig. 3a–c). How can human telomerase use such a short primer–template duplex as the substrate? A prominent feature of human telomerase is that it is an RNP complex whereas canonical RTs are all protein-only enzymes. In the catalytic core of human telomerase, the TERT protein and t-PK domain of hTR are organized in an intercalated double-ring architecture (Fig. 4a). TERT subdomains, TRBD, fingers, palm and thumb, form the inner ring, embracing the short primer–template duplex in the central cavity (Fig. 4a). From the opposite angle, a triangle-shaped RNA ring encompasses the TRBD and thumb subdomains of TERT (Fig. 4a). The intercalated RNA and TERT rings constitute a ~45° dihedral angle, which enlarges the TERT–hTR contact area and buries ~5750 Å^2^ exposed interface area (Fig. 4a). A highly positively-charged patch on the surface of TERT_TRBD_ mediates intensive interactions with the terminal major groove of pseudoknot stem P3 (Fig. 4b; Supplementary information, Fig. S9a). Adjacent to this interface, helix α13 of TERT_TRBD_ sits on the first base pair of stem P1b — TBE of human telomerase, functioning as an anchor to secure P1b in a fixed position on the surface of TERT_TRBD_ (Supplementary information, Fig. S9a). Collectively, these intimate interactions allow TERT_TRBD_ to snugly fit into the junction between stems P1b and P3 (Fig. 4b; Supplementary information, Fig. S9a).

The TWJ domain sits atop of TERT_TRBD_ perpendicular to the t-PK ring (Fig. 4b). TERT_TRBD_ occupies the space between stems P6 and P6.1 with the long helix α21 sticking into the wedge and making close contacts with the highly conserved junction nucleotide A301 (Fig. 4b; Supplementary information, Fig. S9b).^44^ The L-shaped junction is immediately connected to a short 3-bp stem P6c along the helical trajectory of P6a–P6b (Supplementary information, Fig. S9b). A 5-nt internal loop (C248–A252) between stems P6c and P5 guides the RNA to make a ~90° turn in the opposite direction towards the biogenesis module (Fig. 1c; Supplementary information, Fig. S9b). Notably, in some human telomerase complexes, histone H2A–H2B heterodimer stabilizes the TWJ on the opposite side of TERT_TRBD_ via the same surface that interacts with the DNA in nucleosomes (Fig. 4c; Supplementary information, Fig. S9c).^29,45^ The functional significance of H2A–H2B heterodimer in human telomerase awaits future studies.

In addition to TERT_TRBD_, the thumb subdomain also mediates interactions with both t-PK and TWJ. The stem loop P6.1 packs onto one end of the helical bundle of TERT_thumb_ with nucleotide U307 sticking into a deep depression formed by the loop between helices α34 and α35 of TERT_thumb_ (Fig. 4d; Supplementary information, Fig. S9d). Markedly, another uracil U177 flips out of stem P3 and also points into the same depression of TERT_thumb_ but from the opposite direction (Fig. 4d; Supplementary information, Fig. S9d). Together, nucleotides U177 and U307 function as the two arms of a tweezer to tightly secure the position of TERT_thumb_ in the catalytic core (Fig. 4d).

We performed MD simulations to investigate how hTR affects the structure stability of TERT. Simulation trajectories clearly showed that TERT alone exhibited a large fluctuation and cannot stably hold the short primer–template duplex in the active site (Fig. 4e). Association of hTR greatly suppressed this fluctuation (Fig. 4e). In particular, the thumb helix and loop (residues 950–980) that form the basic pocket for binding the DNA backbone were greatly stabilized upon hTR association (Fig. 4e; Supplementary information, Fig. S9e). Moreover, MD simulation also showed that hTR association enhanced the cross-correlation among the subdomains in the TERT ring so that they behave more as a whole in the catalytic core (Fig. 4f). Consequently, in the presence of the hTR RNA, the distances between the zipper head Leu980 and three essential aspartate residues (Asp712, Asp868 and Asp869) at the catalytic center are constrained to optimal values for accommodating short primer–template duplex substrates (Fig. 4g, h). In aggregate, our structural and MD simulation results suggest that the hTR RNA stabilizes TERT, enabling a competent, unique reverse transcriptase with a preformed active site that can catalyze telomere extension from a short primer–template hybrid. Consistently, three-dimensional mapping of disease-derived mutations onto the corresponding positions in hTR unveils that many of them are spatially clustered in the t-PK and TWJ domains of the RNA (Supplementary information, Fig. S9f), presumably resulting in incompetent conformation of human telomerase.

### Double-fingers architecture in TERT facilitates NAP

**Fig. 5:**
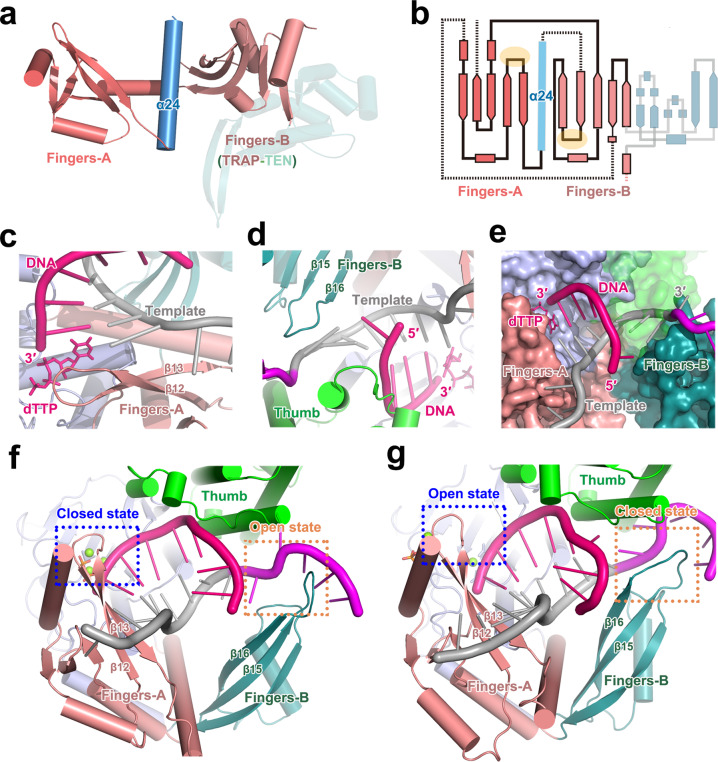
Double-fingers architecture of TERT facilitates NAP. **a** Structural similarity between fingers-A and fingers-B. Fingers-A and fingers-B are colored in deep salmon and salmon, respectively. **b** Topology diagram of the fingers-A and fingers-B subdomains. **c**, **d** Functional parallelism of fingers-A and fingers-B. Fingers-A separates the template strand from the DNA substrate (**c**), while fingers-B separates the product strand from the RNA template (**d**). A dTTP nucleotide was modeled at DNA position 1. **e** The pseudo-two-fold symmetric double-fingers architecture limits the number of template nucleotides in the active site. **f**, **g** Conformational changes of fingers-A and fingers-B before (**f**) and after (**g**) the pyrophosphate dissociation revealed by meta-dynamic simulations. The hairpins β12–β13 in fingers-A and β15–β16 in fingers-B are highlighted with dashed blue and orange boxes, respectively.

Similar to the TRAP motif identified in *T. thermophila* TERT, the large insertion in the fingers subdomain of TERT (residues 738–800) folds into a motif with three β strands and an α helix (Fig. 5a).^6^ This motif mediates close contacts with the N-terminal TEN subdomain by forming a seven-stranded β sheet and burying ~960-Å^2^ exposed interface area (Fig. 5a). Therefore, TEN and TRAP constitute a unique TEN–TRAP subdomain structurally independent of the rest of TERT (Fig. 5a). In accordance with this idea, cross-correlation analysis showed that the dynamic movement of TRAP is correlated with TEN but not the rest of TERT (Fig. 4f).

Close examination of the architecture of the TEN–TRAP and the fingers subdomains unveils a striking similarity — the structural cores of both subdomains are composed of a central multi-stranded β sheet, forming a two-fold pseudo-symmetry along an axis perpendicular to helix α24 of TERT (Fig. 5b). In addition to this structural similarity, the fingers and TEN–TRAP subdomains also display a functional parallelism. In the fingers subdomain, the long hairpin β12–β13 in the β sheet separates the template strand from the DNA substrate and stabilizes the terminal DNA nucleotide at the catalytic center (Fig. 5c). On the other side of the primer–template duplex, the equivalent hairpin β15–β16 in TEN–TRAP separates the product strand from the RNA template, and secures the flanking RNA 5′ to the template on the surface of the thumb subdomain (Fig. 5d). To emphasize these structural and functional similarities, we renamed fingers and TEN–TRAP as the fingers-A and fingers-B subdomains of TERT (Figs. 4a, 5a). This unique pseudo-two-fold symmetric double-fingers architecture can only allow at most seven template nucleotides in the active site (Fig. 5e), suggesting that the fingers-B subdomain is specifically evolved in TERT to meet the requirement for repeated alignment with a short telomere template during telomere extension.

Previous structural studies of HIV-RT revealed that the fingers subdomain undergoes a large conformational change between open and closed states; binding of the correct dNTP at position 1 results in fingers closure, while dissociation of the product pyrophosphate induces fingers opening.^46,47^ To investigate the roles of fingers-A and -B in human telomerase, we monitored their conformational changes during nucleotide addition process by meta-dynamic simulations. Consistent with HIV-RT, removal of the product pyrophosphate from the catalytic center initiates the translocation process of the primer–template duplex and induces an opening movement of hairpin β12–β13, which stabilizes the dNTP at position 1 in the closed state (Fig. 5f, g). Strikingly, principle component analysis (PCA) showed that the opening of hairpin β12–β13 in fingers-A is coupled with a closure of hairpin β15–β16 in fingers-B onto 5′-flanking ss RNA (Fig. 5f, g; Supplementary information, Video S1). This coupled seesaw-like movement of fingers-A and -B implies a mechanism of how human telomerase ensures NAP during telomere extension. When fingers-A closes down to the catalytic center to facilitate the nucleotidyl-transfer reaction, fingers-B opens up to prepare for duplex translocation (Fig. 5f, g). After catalysis, pyrophosphate release induces fingers-A opening, duplex translocation and the closure of fingers-B, so that after translocation fingers-B clamps the template strand on TERT_thumb_ to stabilize the primed duplex substrate that has only three canonical and one wobble base pair in the active site ready for another round of nucleotide addition (Fig. 5f, g).

Notably, the dynamic movement of fingers-B has also been proposed to play an important role in *T. thermophila* telomerase action.^28^ However, in contrast to our seesaw-like movement of fingers-B in each step of nucleotide addition of human telomerase, the *T. thermophila* telomerase study proposed that fingers-B is only in the open state to allow RNA template translocation at the end of the telomere extension cycle.^28^ Future studies are needed to investigate and verify these two models.

### Zipper head promotes RAP

The zipper head residue Leu980 in the thumb helix of TERT limits the number of W-C base pairs of the primer–template duplex during telomere extension, hence representing a disadvantage for the NAP of human telomerase (Fig. 3b). On the contrary, this zipper head is an advantage for RAP; the short duplex could facilitate primer–template separation before template RNA translocation at the end of each telomere extension cycle. Therefore, we conclude that the zipper head residue in TERT provides a subtle mechanism to maintain a limited primer–template duplex that is stable enough for NAP and short enough for RAP.

**Fig. 6:**
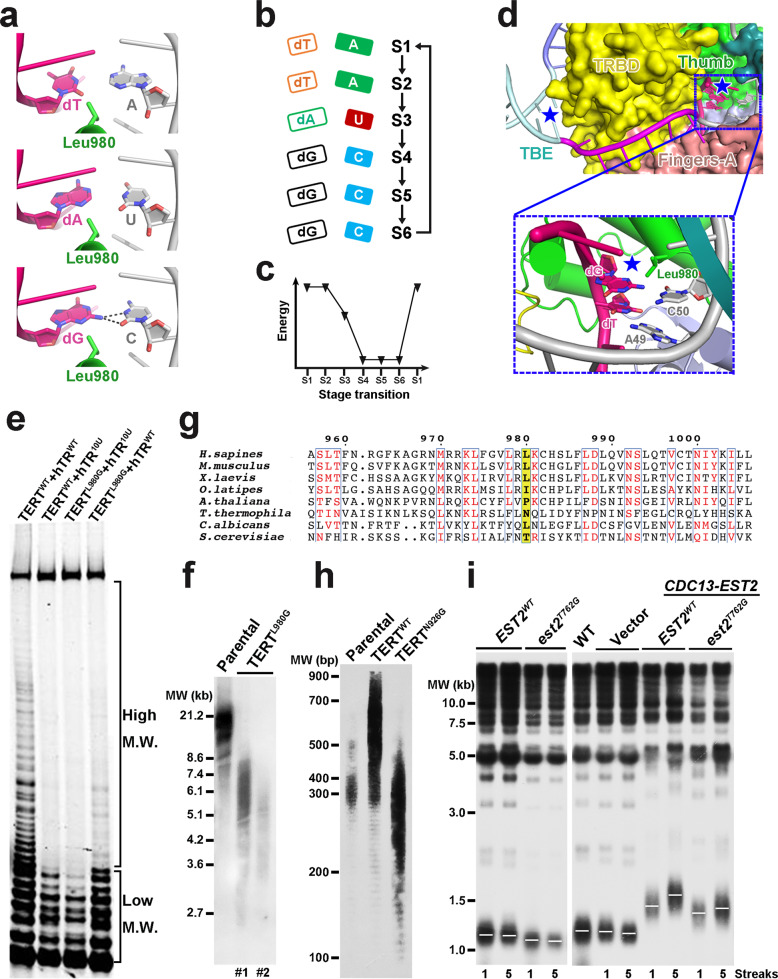
Zipper head Leu980 promotes RAP. **a** Local geometry around Leu980 and adjacent nucleotides when Leu980 encounters dT–A, dA–U, and dG–C base pairs during telomere synthesis cycle. Hydrogen bonds are shown in dashed gray lines. **b** Schematic diagram of sequential base pairs that encounter zipper head Leu980 during a full extension cycle of a telomere repeat. **c** Schematic free energy diagram for zipper head Leu980 against different base pairs during the sequential transition as in **b**. **d** Zipper head Leu980 coordinates TBE to define the template boundary at the end of each telomere extension cycle. The zipper head- and TBE-defined pausing signals are designated with blue stars. The inset shows a close-up view of dT and dG at DNA positions 4 and 5 facing the zipper head residue Leu980 at the active site at the end of each cycle. **e** Activity of human telomerase purified from U2OS cells co-expressing the indicated hTR and Flag-tagged TERT. **f** Southern blot measurement of telomere restriction fragment (TRF) lengths for 293T parental and two TERT^L980G^ knock-in clonal cell lines after ~20 population doublings, denoted as #1 and #2. **g** Multiple sequence alignment of the thumb helix flanking sequence of TERT proteins from various eukaryotes. Positions equivalent to human TERT^Leu980^ are highlighted in yellow. **h** Southern blot measurement of TRF lengths for *T. thermophila* cells overexpressing WT or N926G mutant TERT. **i** Est2^Thr762^ residue is required for telomere maintenance in *S. cerevisiae*. Left: Southern blot analysis of telomere lengths of *EST2*^*WT*^ and *est2*^*T762G*^ cells. Right: Est2^T762G^ mutation affected the telomere lengthening caused by Cdc13–Est2 fusion. Genomic DNAs extracted from the 1st and 5th cultures were digested with *Xho* I and subjected to Southern blot analysis with a telomere-specific TG_1–3_ probe.

During human telomere extension, zipper head residue Leu980 encounters three different types of base pairs at position 5, dT–A, dA–U and dG–C (Fig. 6a). To examine whether the zipper head could distinguish these base pairs, we focused on the local geometry around Leu980 and nucleotides dA5 and U53. The hydrophobic methyl group of Leu980 is only ~3.0 Å away from the imino group of dA5, resulting in an energetically unfavorable contact that pushes the dA5–U53 pair towards the major groove of the primer–template duplex (Fig. 6a). Structural modeling unveiled that the methyl group of Leu980 will have a close contact with a similar imino group of guanine when a dG–C pair is at position 5 (Fig. 6a). It is noteworthy that a dG–C pair could still potentially maintain hydrogen bonds even it is in a distorted conformation, indicating that a dG–C pair is likely more favorable than dA–U at this position (Fig. 6a). In contrast, a dT–A pair at position 5 would generate the most unfavorable situation where the methyl group of Leu980 has to face a carbonyl oxygen of thymine, which is more negatively charged than an imino group (Fig. 6a). Consistently, thermodynamic integration free energy calculations showed that zipper head Leu980 would most favor dG–C, then dA–U and last dT–A pairs at position 5, supporting the notion that zipper head Leu980 can read the nucleotide identity at position 5 (Supplementary information, Fig. S10a).

During a full extension cycle of a telomere repeat starting from the 3′ alignment region, Leu980 sequentially encounters two dT–A, one dA–U, and three dG–C base pairs (Fig. 6b). Notably, this sequence represents an energetically downhill process from the least to the most favorable base pairs at position 5 for Leu980, in accordance with previous data that once the first deoxyguanylate is successfully incorporated, the rest five nucleotides will be rapidly incorporated to complete a cycle of telomere extension (Fig. 6b, c).^22,48^ At the end of each cycle, DNA positions 4 and 5 at the active site are occupied by dT and dG, respectively (Fig. 6d). Even if the primer–template duplex could translocate for another cycle, the zipper head Leu980 would experience an unfavorable dG-to-dT transition at position 5, potentially leading to an arrest during translocation (Fig. 6c, d). Indeed, this zipper head-based arresting site is in perfect agreement with the sequence-defined pausing signal of human telomerase revealed by previous biochemical studies (Supplementary information, Fig. S10b),^23^ suggesting that the zipper head residue Leu980 is likely the structural determinant responsible for reading the sequence-based template boundary signal (Fig. 6b, c). Taken together, we propose that the zipper head residue Leu980 plays two different roles in promoting RAP of human telomerase: (1) limiting the number of the W-C pairs of the duplex to facilitate primer–template separation before template RNA translocation (Fig. 3); (2) defining a sequence-based template boundary that coincides with the TBE-defined boundary (Fig. 6d; Supplementary information, Fig. S10b).

To corroborate our structural analysis, we performed telomere repeat amplification (TRAP) assay to investigate the role of zipper head Leu980 in human telomerase activity. The TRAP result showed that glycine substitution of Leu980 substantially reduced the high molecular weight products but only had a marginal effect on low molecular weight products, demonstrating that Leu980 plays a crucial role in promoting RAP (Fig. 6e). To further address the in vivo function of the zipper head, we examined the effect of the L980G mutation on telomere length maintenance. Southern blot analysis showed that, after ~20 population doublings, mutant cells exhibited highly heterogeneous and short telomeres compared to wild-type (WT) cells (Fig. 6f). We conclude that the RAP-promoting function of the zipper head is essential for telomere homeostasis (Fig. 6e, f).

In addition to the zipper head residue, alanine substitutions of positively-charged amino acids (Lys973 and Lys981) in the thumb helix also resulted in decreased RAP (Supplementary information, Fig. S10c, d). These residues contribute to correct positioning of the zipper head Leu980 in the active site to stick into the duplex minor groove to limit the duplex length (Fig. 3a). In accordance with the importance of TBE in promoting RAP,^19–21^ disruption of the TBE by insertion of 10 uridylates between nucleotides 38 and 39 of hTR (hTR^10U^) greatly decreased telomerase RAP (Fig. 6e). Notably, double mutation of TERT^L980G^/hTR^10U^ only slightly further reduced the RAP compared to the single mutations (Fig. 6e). Collectively, these data suggest that the zipper head and TBE together function as a double-safe mechanism to assure the template translocation step at the end of each telomere extension cycle to facilitate RAP.

### Conservation of the zipper head mechanism

The important role of the zipper head residue Leu980 in promoting RAP of human telomerase promoted us to ask whether this zipper head mechanism is universally conserved in eukaryotes. In regular protein-only RTs, the invariant glycine residue equivalent to human TERT^Leu980^ serves as an important modulator of both processivity and fidelity, so that even an extra methyl group in the Gly-to-Ala mutation leads to elevated pausing during DNA synthesis.^40^ In sharp contrast, positions equivalent to human TERT^Leu980^ in all TERT proteins are occupied by non-glycine amino acids, leucine or isoleucine in vertebrates, proline in plants and asparagine in ciliates (Fig. 6g; Supplementary information, Fig. S11a). Concordantly, structural comparison revealed that the active sites in *T. thermophila* and *Candida albicans* TERT proteins are identical to that in human TERT with zipper head residues Asn926 in *T. thermophila* TERT and Leu766 in *Candida albicans* TERT corresponding to Leu980 in human TERT (Supplementary information, Fig. S11b).^28,29,49^ Similar to human TERT^Leu980^, these non-glycine zipper head residues very likely also limit the length of the primer–template duplex in the active site and facilitate RAP during repetitive telomere extension. Therefore, we propose an evolutionally conserved zipper head mechanism for all eukaryotic telomerases in which a non-glycine residue, not its amino acid identity at the zipper head position of TERT, is essential for the repetitive telomere extension.

To test this idea, we investigated the functional importance of human TERT^Leu980^ equivalent residues *T. thermophila* TERT^Asn926^ and *S. cerevisiae* Est2^Thr762^ in telomere length homeostasis (Fig. 6g). Overexpression of WT TERT induced rapid telomere lengthening in *T. thermophila* cells (Fig. 6h). In contrast, the TERT^N926G^ mutation greatly reduced over-extended telomeres compared to WT TERT (Fig. 6h). Similarly, a glycine substitution of Est2^Thr762^ led to shortened telomeres in *S. cerevisiae* cells (Fig. 6i). The direct fusion of Est2 to Cdc13 has been previously shown to give rise to telomere over-elongation.^50^ Notably, the Est2^T762G^ mutation substantially reduced the telomere lengthening caused by the Cdc13–Est2 fusion (Fig. 6i). Taken together, our results demonstrated that the zipper head residues (human TERT^L980^, *T. thermophila* TERT^N926^ and *S. cerevisiae* Est2^Thr762^) contribute to telomere length homeostasis in all three different organisms, supporting the conservation of the zipper head mechanism in eukaryotic telomerases.

Similar to our structure, a short primer–template duplex in the active site was also observed in the recent structures of human and *T. thermophila* telomerases.^28,29^ A bridge loop in *T. thermophila* TERT_RBD_ was proposed to help maintain this short duplex through a stacking interaction between TERT^Phe414^ and product nucleotides at positions 5–8 that flip away from the duplex.^28^ We propose that the zipper head residue Asn926 in *T. thermophila* telomerase plays a major role in limiting the duplex length to separate the template from the primer strand beyond position 4, and the latter is then stabilized by the bridge loop. Structural comparison revealed a corresponding bridge loop in our and the recently reported human telomerase structures (Supplementary information, Fig. S10c). However, it does not mediate any contact with the substrate DNA (Supplementary information, Fig. S10c). Whether the bridge loop plays a similar role in stabilizing nucleotides outside of the primer–template duplex in telomerases of human and other species awaits future studies.

## Discussion

### A model of telomere synthesis by human telomerase

**Fig. 7:**
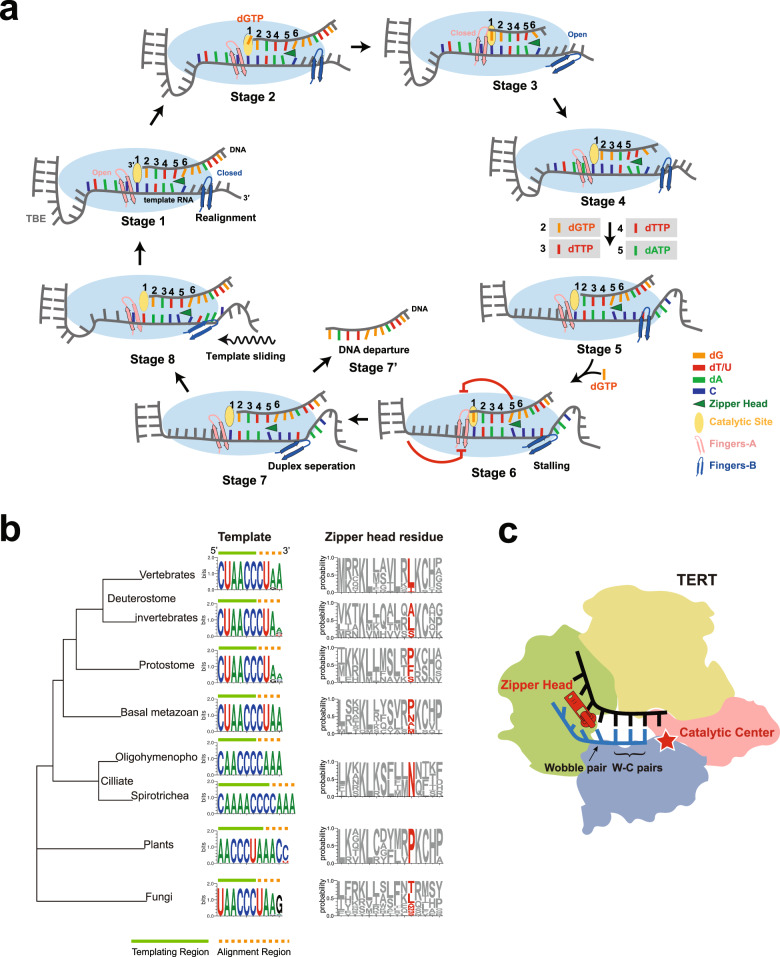
A model of telomere synthesis by human telomerase and coevolution of telomerase RNA template sequence and the zipper head mechanism. **a** A schematic model for telomerase catalytic cycle of repeat synthesis. At the beginning of each telomerase catalytic cycle (Stage 1), base pairing of telomeric repeat with the template alignment region forms a duplex with three W-C and one wobble base pairs at positions 2–5, which fits into the active site defined by the zipper head residue Leu980. Fingers-A opens up for the binding of an incoming dNTP, while the closure of fingers-B stabilizes the primed duplex substrate. After incorporation of dGTP into position 1, fingers-A closes down to facilitate the nucleotidyl-transfer reaction, and at the same time fingers-B opens up from the template strand for nucleotide translocation (Stages 2 and 3). Subsequently, the two fingers undergo conformational changes back to the ‘primed state’ for the next round of nucleotide addition (Stage 4). After completion of synthesis of one telomere repeat with five consecutive nucleotide incorporation (Stage 5), a double-safe pausing mechanism defined by both the zipper head and the TBE stem P1b stalls DNA synthesis (Stage 6). After unpairing of the stalled primer–template duplex (Stage 7), telomerase either falls off from the telomere end (Stage 7’), or experiences template translocation back to the original position and primer–template realignment for another round of repeat synthesis (Stages 8 and 1). **b** Coevolution of telomerase RNA template sequence and the zipper head mechanism. Left panel: simplified phylogenetic tree of eukaryotic lineages. Middle panel: logo representations of telomerase RNA template sequences from 42 vertebrates, 30 invertebrates in deuterostome, 26 protostome, 27 basal metazoan, 31 fungi with vertebrate type telomeric repeat and 15 plants. Right panel: logo representations of thumb helix flanking sequences of TERT proteins from species as in the Middle panel. Zipper head residues are highlighted in red. **c** Schematic model of the evolutionally conserved zipper head mechanism in telomerase. The zipper head, catalytic center, W-C and wobble base pairs are denoted.

Based on our structural and computational data reported here combined with previous studies, we propose an integrated model for human telomerase-mediated telomere synthesis (Fig. 7a). At the beginning of each cycle, the 3′ terminus of telomeric overhang pairs with the 3′ alignment region of the RNA template to form a duplex with three W-C and one wobble base pairs at positions 2–5, which fits into the active site defined by the zipper head residue Leu980 (Fig. 7a, Stage 1). In this ‘primed’ state, fingers-B clamps the 3′ template region on TERT_thumb_ to stabilize the duplex substrate, while fingers-A opens up from the catalytic center for the binding of an incoming dNTP (Fig. 7a, Stages 1 and 2). Upon the incorporation of a correct dGTP into position 1, fingers-A closes down to the catalytic center to facilitate the nucleotidyl-transfer reaction, and at the same time fingers-B opens up from the template strand (Fig. 7a, Stage 3). Release of the pyrophosphate product powers the translocation of the primer–template duplex and the conformational change of the two fingers back to the ‘primed’ state, ready for the next round of nucleotide addition (Fig. 7a, Stage 4). After five consecutive nucleotide incorporation (Fig. 7a, Stage 5), the double-safe mechanism defined by both zipper head Leu980 and the TBE stem P1b stalls the telomere end at the 5′ terminus of the RNA template (Fig. 7a, Stage 6). We propose that the release of the pyrophosphate product from the last dGTP incorporation induces a cascade of coupled events — unpairing of the stalled primer–template duplex, retention of the telomere end in the active site by electrostatic interactions with the thumb helix and loop, and sliding of the template RNA through the telomere DNA back to the position at the beginning of the synthesis cycle — to prepare for another round of telomere extension (Fig. 7a, Stages 7 and 8). During the translocation process, telomerase may also fall off from the telomere end to terminate the telomere synthesis (Fig. 7a, Stage 7’). Finally, translocated 5′ alignment region of the RNA template realigns with the last four nucleotides TTAG at the telomere end to reform the short primer–template duplex that rebinds into the active site to initiate the next round of repeat synthesis (Fig. 7a, Stage 1). Template translocation and the realignment of the primer–template duplex accompany large-scale conformational changes, in accordance with the fact that the first nucleotide incorporation following template translocation is the rate-limiting step for processive repeat addition.^22,48^ Future studies are required to fully understand the mechanism of how human telomerase achieves this complicated and highly orchestrated process.

### Coevolution of telomerase RNA template and the zipper head mechanism

Phylogenetic analysis revealed that the template region is the most conserved fragment in telomerase RNA components, with the consecutive cytosine nucleotides always in the center of the RNA template region (Fig. 7b).^51,52^ This unique feature of the RNA template implies that telomerase RNA has coevolved with the zipper head residue to assure efficient RAP during telomere extension. At the end of each cycle of repeat synthesis after dNTP incorporation, the zipper head residue only allows a wobble and four canonical W-C base pairs to be maintained at the 5′ end of the RNA template in the active site of telomerase, among which there are at least two less stable A–T/U pairs to facilitate duplex separation before template translocation (Fig. 7a, Stage 6). This exquisite collaboration between the RNA template sequence and the zipper head residue indicates that these unique telomerase elements might had coevolved to ensure an efficient RAP in telomere synthesis since the early stage of eukaryotic evolution.

Another salient feature of the telomerase RNA template is that the 3′ alignment region is less conserved than the 5′ template region, and in many cases the 3′ terminal nucleotide cannot form the canonical W-C base pair with the telomere DNA (Fig. 7b). Structurally, this 3′ terminal nucleotide at the alignment region is always at position 5 of the RNA template strand and mediates a wobble base pair with the DNA during the primer–template realignment step (Fig. 7a, Stage 1). This functional feature explains why the 3′ terminus is the least stringent position in the RNA template and provides another evidence of the coevolution between the zipper head mechanism and the RNA template sequence of telomerases.

In summary, our structural and computational studies of human telomerase holoenzyme with bound telomeric DNA provide novel insights into the molecular basis of repetitive telomere synthesis. In particular, we reveal a conserved zipper head mechanism that limits the length of the short primer–template duplex in the active site and facilitates RAP of human telomerase (Fig. 7c). Coevolution of telomerase RNA template sequence and the zipper head mechanism balances NAP and RAP efficiencies to ensure both processivity and fidelity of telomere repeat synthesis.

## Materials and methods

### Cell line establishment

To prepare human telomerase RNP complex for structural studies, we established a stable cell line to overexpress TCAB1. TCAB1 was cloned into a modified lentivirus expression vector pLVX-IRES-EGFP with an N-terminal twin-strep tag. The constructed lentiviral expression vectors together with the virus packaging plasmids were introduced into 293T cells using x-tremeGENE HP transfection reagent (Roche) according to the manufacturer’s recommendations. After 48-h transfection, the virus-containing supernatants derived from these 293T cells were used to infect the Expi293F cells (Thermo Fisher, A14527). After 12-h infection, the cells were replaced with fresh medium. Three days after infection, the infected cells were subjected to flow cytometry to sort out the EGFP-positive TCAB1-expressing cells, referred to as Tc6-3 cells. The 293T cells were cultured in DMEM supplemented with 10% FBS, while the Expi293F cells were maintained in chemically defined 293 medium (Cell-Wise, BWCXA019).

### Expression and purification of human telomerase holoenzyme

The human telomerase holoenzyme was reconstituted in Tc6-3 cells transiently transfected with hTERT-expressing plasmid DNA (pcDNA3.4-triple Flag-TERT) and hTR-expressing plasmid DNA (pcDNA3.1-U3-hTR-HDV). HDV is the hepatitis delta virus ribozyme, which was previously shown to increase hTR accumulation.^53,54^ The two plasmids were introduced into Tc6-3 cells using polyethylenimine (PEI, Polysciences, 24765), after ~48 h cells were harvested for purification. Approximately 8 L of Tc6-3 cells with transient overexpression of TERT and hTR at a density of ~3.0 × 10^6^ cells/mL were collected by centrifugation at 3000× *g* for 15 min. The collected cell pellets were washed once with PBS and then resuspended in buffer A (20 mM HEPES, pH 7.9, 200 mM KCl, 10% glycerol, 2 mM MgCl_2_, 1 mM EDTA, 0.1% IgePal CA-630) with protease inhibitor cocktails (Roche). The suspended cells were frozen into beads with a diameter of ~5 mm using liquid nitrogen. The cell beads were broken down in a SPEX 6870D Freezer Mill. The cell lysate was cleared by centrifugation at 20,000× *g* for 50 min. The supernatant was applied to Strep-Tactin XT Superflow high-capacity resin (IBA Lifesciences, 2-4030-010) for ~3 h. The resin was washed with 150 mL buffer A to remove the contaminant. The human telomerase holoenzyme was eluted with 40 mL of buffer B (20 mM HEPES, pH 7.9, 200 mM KCl, 10% glycerol, 2 mM MgCl_2_, 1 mM EDTA) supplemented with 50 mM biotin (Sigma, B4501), 1 mM DTT and 100 U/mL RNase Inhibitor (Thermo Fisher Scientific). The eluent was then incubated with anti-Flag resin (GenScript Biotech, L00425) overnight. After washing the resin with 50 mL buffer A, the human telomerase holoenzyme  was eluted with buffer C (20 mM HEPES, pH 7.9, 150 mM KCl, 1 mM MgCl_2_) containing 0.2 μg/μL Flag peptide for electron microscopy study immediately. To prepare DNA substrate-bound telomerase sample, oligonucleotide T_20_AG_3_ (100 nM) was added during the incubation step with  the anti-Flag resin, and excess T_20_AG_3_ (1 μM) was also added before cryo-EM sample preparation.

### Cryo-EM grid preparation and data collection

Cryo-EM grids were prepared with the Vitrobot Mark IV plunger (FEI) set to 8 °C and 100% humidity. Approximately 3 μL of the purified sample was deposited onto the glow discharged Ted Pella lacey copper grids (Ted Pella, 01824; PELCO easiGlow) coated with a thin layer of continuous carbon film. A blot force of −1 and blot time of 3 s were applied to blot the grids after incubation of the sample with grids for 10 s. Then the samples on grid were vitrified by plunge freezing in pre-cooled liquid ethane at a liquid nitrogen temperature.

All micrographs were acquired on a FEI Titan Krios G3i operated at 300 keV, equipped with Gatan K3 direct electron detector. Automated image acquisition was performed with EPU Software (FEI Eindhoven, the Netherlands) at a magnification of 81,000×, corresponding to a pixel size 0.55 Å. The K3 detector was gain-corrected and micrographs were collected at a defocus varying between −1.5 μm and −2.8 μm. We collected a total of 40 frames, amounting to a total dose of 62 e^–^/Å^2^ over 3.0 s of exposure.

### Cryo-EM data processing

Particles were automatically picked with Gautomatch, using templates of 2D averages obtained from the previous negatively stained dataset. For each dataset, all particles were normalized and binned by 4 during the extraction with a box size of 104^2^ pixels and pre-aligned using multiple rounds of 2D classification in RELION 3.1.^55^ All classes of good quality were selected and re-extracted to bin 1 and joined together, resulting in a dataset of a total number of 771,617 particles. To deal with the intrinsic conformational heterogeneity, the data were then treated as consisting of two relatively independent parts, the catalytic core and the biogenesis module, by using focused classifications and recentering.

For the catalytic core, a focused classification based on global search was performed with 7 classes and the regularization parameter T = 60 by applying a tight mask on the corresponding part in the reference structure. The best class was selected and recentered, and then further refined to 3.54 Å. In the meantime, a 3D classification without alignment was also done to the same set of particles based on the prior orientation obtained from the previously focused classification. The class of data showing clear density of H2A/H2B was further refined to 4.97 Å.

The same type of focused classification was done with 12 classes and the regularization parameter T = 40, by using a mask covering the biogenesis module in the reference. The particles of the highest quality were selected, and then refined to 3.94 Å after recentering. Moreover, to reveal clear density of the RNA between stems P7b and P8a, the same set of particles were classified into 6 classes without alignment, by masking the corresponding local area in reference. The set of particles with 3D class average showing the clear density of interest were further re-centered and refined to 4.40 Å.

### Model building

We combined de novo model building and rigid-body docking of components with known structures to generate the atomic model of human telomerase holoenzyme. The model was then manually built and adjusted in Coot.^56^ The final model refinement was carried out using phenix.real_space_refine^57^ with secondary structure and geometry restrains to prevent over-fitting. The structure of human telomerase holoenzyme was validated by Molprobity.^58^ Figures of the EM density maps and the structural model were generated using PyMol or UCSF Chimera.^59^

### MD simulations

All systems were set up using AmberTool. The amber ff14sb^60^ was used for proteins and the revised RNA. ROC^61^ force field and the DNA OL15^62^ were used for RNA and DNA, respectively. The TIP4PEW water model was used to solvate the system with a buffering distance of 1.2 nm and magnisium ions with 12-6-4 parameter^63^ set was used to neutralize the charges of system, which yields a whole simulation system with a total of 293,938 atoms in a starting dimension of 135.75 Å × 143.00 Å × 138.38 Å. The system was initially refined using 500 steepest descent steps before switching to conjugate gradient energy minimization and gradually heated to 300 K within 2 ns. The positional restraints were exerted on the backbone of the protein, RNA and DNA molecules with a weight of 10 kcal/mol/Å^2^ during the energy minimization and heating process. Then the restraints were released gradually within six equilibration steps under constant pressure and temperature (NPT) ensemble. The hydrogen mass repartitioning was set to 4 amu to enable an integration step of 4 fs for the simulations. Simulations were conducted using a 12 Å cutoff distance for the Lennard-Jones interactions and the Particle Mesh Ewald (PME) summation method was used for calculating Coulomb interactions. MD simulation system by removing the hTR RNA was also constructed to investigate how hTR affects the structure stability of TERT and comparative MD simulations of Leu980Gly mutation were also performed. For each simulation system, 5 × 500 ns MD trajectories were generated for further analyses.

Thermodynamic integration (TI) method^64^ was used to investigate the free energy changes by decoupling the side chain of Leu980 facing dG–C, dA–U and dT–A at position 5, respectively. The Leu980Gly simulation system was constructed using the AMBER dual-topology TI framework. The similar equilibration steps were performed as regular simulations but using the coupling parameter λ of 0.5. Then 21 perturbing parameters from 0 to 1 with equal increment of 0.05 were used for TI simulations with soft-core potentials implemented with the GPU-accelerated version of PMEMD, the production runs for each coupling parameter were conducted for 20 ns, and then trapezoidal rule integration formulas were used for evaluating the free energy changes during the alchemical transformation.

We also performed the well-tempered meta-dynamic simulations^65^ to investigate the pyrophosphate dissociation process after the nucleotide addition is completed. The parameters of pyrophosphate group were prepared using the GAFF force field.^66^ The Gaussian 09 package^67^ with B3LYP/6-31 G* method was employed to perform the geometry optimization of compounds and the restrained electrostatic potential (RESP) approach^68^ was used to assign the partial charges of pyrophosphate. The OpenMM package^69^ through GPU acceleration combining with PLUMED^70^ enhanced-sampling library was employed to perform the metadynamic simulations. The Langevin Integrator was used with a collision frequency of 1.0/ps to couple the system’s temperature at 300 K. The PME and the dispersion correction algorithm were exploited to estimate the contribution of long-range non-bonded interactions beyond the cutoff of 12 Å. The distance between the center of mass of the pyrophosphate leaving group and the newly formed phosphate was used as collective variable (CV) to drive the dissociation process. A bias factor of 35 was used and the Gaussian widths were set to 0.1 Å with the height of 2.0 kJ/mol added by every 1000 steps. A distance threshold of 25 Å was set to terminate the simulation after the pyrophosphate was released from the reaction center. Five independent well-tempered meta-dynamic simulations were performed and the pyrophosphate dissociation process could be observed within 50 ns of our simulations.

The analysis of the RNA–DNA conformational dynamics along our simulation trajectories was performed using CURVES+,^71^ which defines the geometrical descriptors of base pair by defining the complementarity parameters such as shear, stretch, stagger, buckle, propeller and opening.

### TRAP

Human telomerase was purified from U2OS cells co-expressing WT and mutant Flag-tagged TERT and hTR. PCR-based TRAP assay was performed as previously described.^72^ Briefly, the reaction was carried out in a 25-μL volume containing 200 ng protein, cy3-labeled TS primer (5′-AATCCGTCGAGCAGAGTT-3′), ACX primer (5′-GCGCGGCTTACCCTTACCCTTACCCTAACC-3′), TSNT primer (5′-AATCCGTCGAGCAGAGTTAAAAGGCCGAGAAGCGAT-3′), NT primer (5′- ATCGCTTCTCGGCCTTTT-3′) and 1 μL Taq^TM^ (TAKARA). The PCR condition was 40 min at 20 °C for an initial extension, 5 min at 95 °C for a hot start, followed by 29 cycles of 30 s at 95 °C for denaturation, 30 s at 55 °C for annealing and 40 s at 72 °C for extension. The PCR products were resolved by 12% polyacrylamide gel electrophoresis at room temperature, and the gel was scanned by a Typhoon scanner (GE Healthcare).

### TRF analysis

The 293T cells were cultured in DMEM supplemented with 10% FBS. The CRISPR-Cas9 mediated genome editing^73^ was performed to generate the TERT^L980G^ knock-in cells by co-transfection of the pX330 plasmid encoding a Cas9 protein and a sgRNA (GCAAACTCTTTGGGGTCTTG), as well as an ss donor DNA. Single clones were obtained by cell sorting, and TERT^L980G^ mutations were confirmed by sequencing. Genomic DNA was purified using a Blood & Cell Culture DNA Mini Kit (Qiagen). TRF analysis was performed using TeloTAGGG Telomere Length Assay (Roche). Briefly, 10 μg of genomic DNA was digested with *Hinf* I/*Rsa* I at 37 °C overnight, and fractionated by electrophoresis on 0.8% agarose gel. The DNA fragments were transferred to a Hybond-N^+^ Nylon membrane (GE Healthcare), and then probe labeling, hybridization and immunological detection were performed following the manufacturer’s instructions.

### Yeast complementation assay

*S. cerevisiae* strain BY4741 was from EUROSCARF (SRD GmbH). To construct in-frame Cdc13–Est2 fusion protein, Cdc13 and Est2 sequences were amplified, and purified Cdc13 and Est2 sequences were used for a second PCR to obtain the full-length Cdc13–Est2 sequence, which was sequentially cloned into plasmid pRS316. PCR-based point mutation of Est2^Thr762^ was made from plasmid covering WT *EST2* (pRS315-*EST2* and pRS316-*CDC13*-*EST2*).

Telomere Southern blot was performed as described previously.^74^ Briefly, cells were harvested from re-streaking plates. Genomic DNA was purified by using phenol chloroform method, digested with *Xho* I, and fractionated by electrophoresis on 1.0% agarose gel. The DNA fragments were transferred to a Hybond-N^+^ Nylon membrane (GE Healthcare), UV cross-linked and incubated with Church buffer for 30 min at 50 °C. Biotinylated telomere-specific probe was incubated with the DNA at 50 °C overnight, and biotin probe-bound DNA fragments corresponding to telomeric DNAs were detected using Chemiluminescent Nucleic Acid Detection Module (Thermo Scientific, USA).

### *Tetrahymena* complementation assay

Transgenes encoding TERT-Flag, TERT(N926G)-Flag were targeted for integration at *BTU1* locus. The *BTU1* promoter and open reading frame were replaced with Flag-tagged TERT under the expression control by ~1 kb of the *MTT1* promoter.^75^ Transformation and taxol selection against the endogenous *BTU1* locus in ciliate was performed as previously described.^76^ Genomic DNA was purified by using phenol chloroform method, digested with *Mse* I, recovered by phenol/chloroform/isoamyl alcohol (25:24:1) extraction and ethanol precipitation, and resolved by denaturing 6% acrylamide/7 M urea/0.6× TBE gel electrophoresis. The DNA fragments were transferred to a Hybond-N^+^ Nylon membrane (GE Healthcare), UV cross-linked and incubated with Church buffer for 30 min at 50 °C. Biotinylated telomere-specific probe (C4A2)3 was incubated with the DNA at 50 °C overnight, and biotin probe-bound DNA fragments corresponding to telomeric DNAs were detected using Chemiluminescent Nucleic Acid Detection Module (Thermo Scientific, USA).

## Supplementary information

Supplementary information, Figure S1

Supplementary information, Figure S2

Supplementary information, Figure S3

Supplementary information, Figure S4

Supplementary information, Figure S5

Supplementary information, Figure S6

Supplementary information, Figure S7

Supplementary information, Figure S8

Supplementary information, Figure S9

Supplementary information, Figure S10

Supplementary information, Figure S11

Supplementary information, Table S1

Supplementary information, Video S1

Supplementary Video Note

## Data Availability

Data supporting the findings of this manuscript are available from the corresponding authors upon reasonable request. The cryo-EM maps have been deposited into the EM Data Bank under accession numbers: EMD-31811 (Catalytic core), EMD-31812 (Histone masked), EMD-31813 (Biogenesis module) and EMDB-31814 (GAR1 masked). Atomic coordinates have been deposited into the Protein Data Bank with PDB codes: 7V99 (Catalytic core) and 7V9A (Biogenesis module).
