# Supplementary material for: Zipper head mechanism of telomere synthesis by human telomerase
Source: Cell Res. 2021 Nov 15;31(12):1275–90. doi: 10.1038/s41422-021-00586-7 (PMC8648750; doi:10.1038/s41422-021-00586-7)
Supplement: Supplementary file 1 — Supplementary information, Figure S1 [file 41422_2021_586_MOESM1_ESM.pdf]

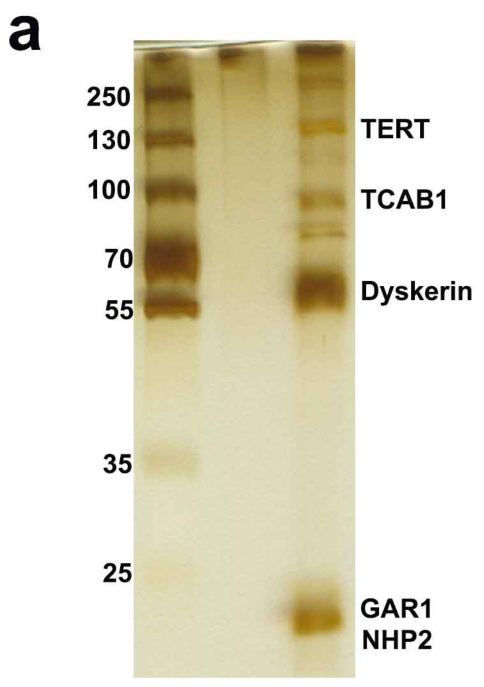

b

Uniprot Acc.No.	*H. sapiens* protein	Peptide spectrum matches	Unique peptides
O14746	TERT	224	64
O60832	Dyskerin	146	41
Q9BUR4	TCAB1	95	22
Q9NY12	GAR1	30	8
Q9NX24	NHP2	26	9
Q9NPE3	NOP10	13	7
O60814	H2B 1-K	15	7
P06899	H2B 1-J	12	6
Q6FI13	H2A 2-A	12	5
⋮	⋮	⋮	⋮
P62805	H4	5	5
P68431	H3.1	2	2

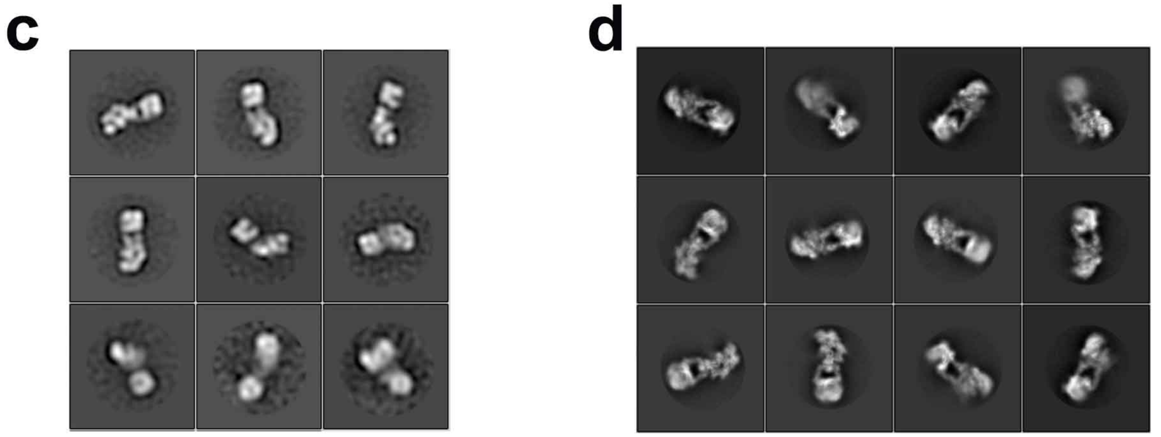

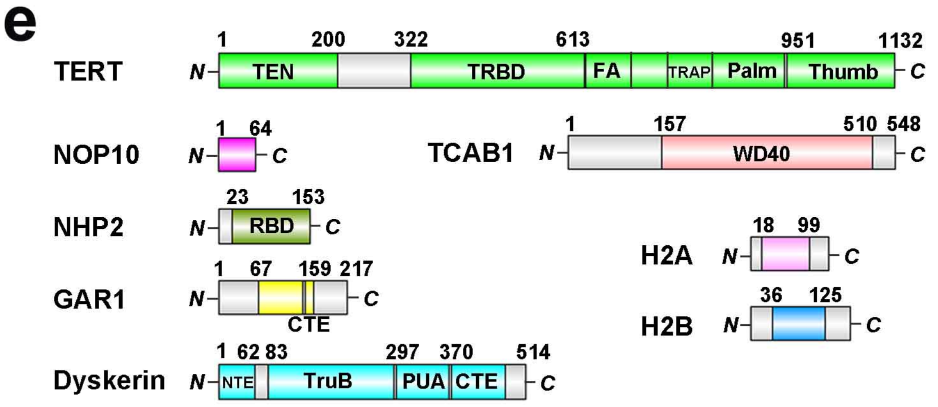

Supplementary information, Fig. S1 Biochemical and biophysical evaluation of purified human telomerase holoenzyme. a Silver-stained SDS-PAGE gel of the purified telomerase holoenzyme complex. **b** Mass spectrometry analysis of the purified telomerase holoenzyme complex. **c** Negative-stained 2D class averages of the purified telomerase holoenzyme complex. **d** Representative 2D class averages of telomerase particles from cryo-EM experiments. **e** Domain organizations of human telomerase protein components.
