# Supplementary material for: Zipper head mechanism of telomere synthesis by human telomerase
Source: Cell Res. 2021 Nov 15;31(12):1275–90. doi: 10.1038/s41422-021-00586-7 (PMC8648750; doi:10.1038/s41422-021-00586-7)
Supplement: Supplementary file 2 — Supplementary information, Figure S2 [file 41422_2021_586_MOESM2_ESM.pdf]

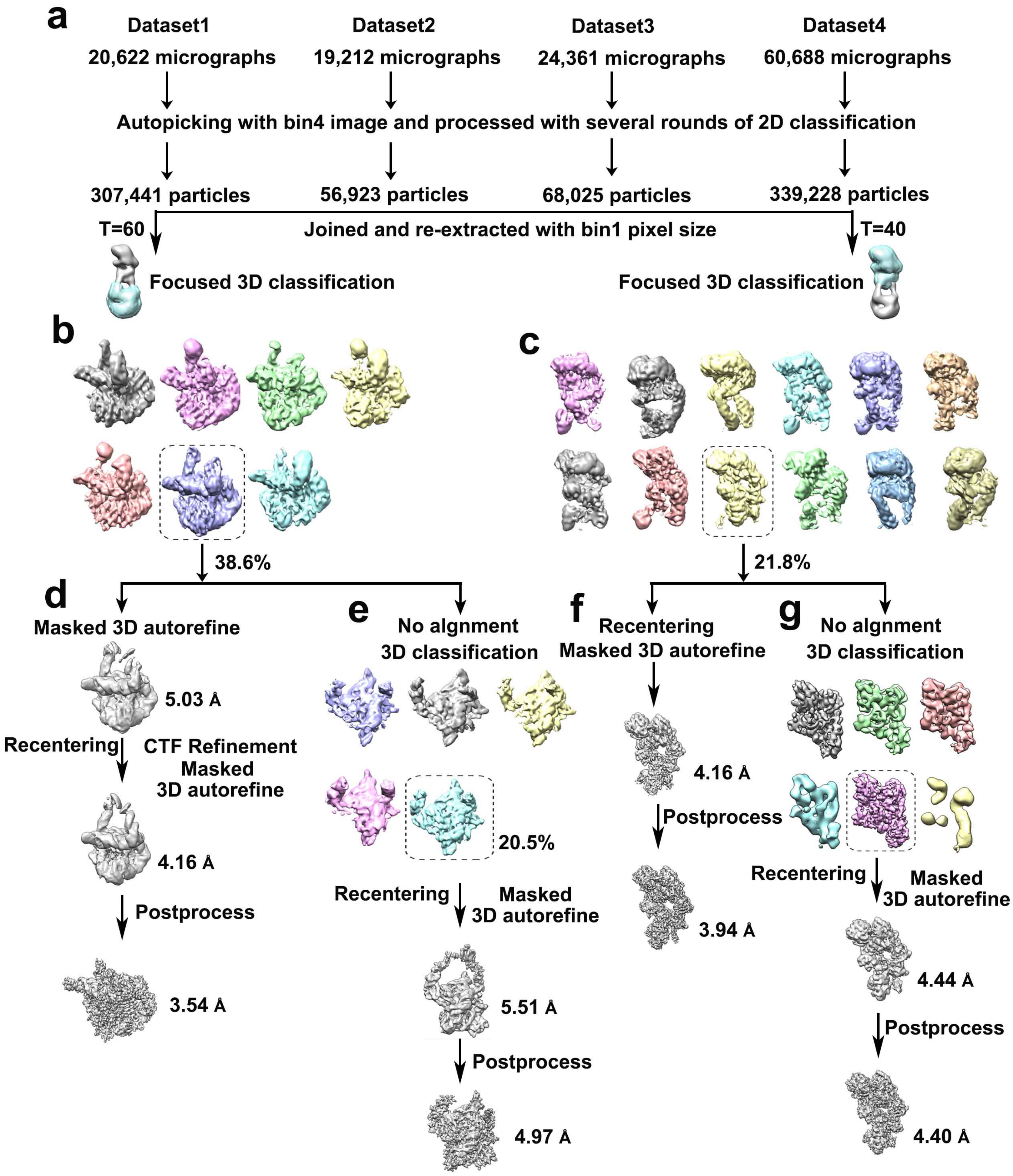
a
Dataset1
20,622 micrographs
Dataset2
19,212 micrographs
Dataset3
24,361 micrographs
Dataset4
60,688 micrographs
Autopicking with bin4 image and processed with several rounds of 2D classification
307,441 particles
56,923 particles
68,025 particles
339,228 particles
T=60
Joined and re-extracted with bin1 pixel size
T=40
Focused 3D classification
Focused 3D classification
b
c
38.6%
21.8%
d
Masked 3D autorefine
5.03 Å
Recentering
CTF Refinement
Masked
3D autorefine
4.16 Å
Postprocess
3.54 Å
e
No algnment
3D classification
20.5%
Recentering
Masked
3D autorefine
5.51 Å
Postprocess
4.97 Å
f
Recentering
Masked 3D autorefine
4.16 Å
Postprocess
3.94 Å
g
No algnment
3D classification
Recentering
Masked
3D autorefine
4.44 Å
Postprocess
4.40 Å

Supplementary information, Fig. S2 Cryo-EM data processing workflow of human telomerase holoenzyme. **a** All datasets and particles used in this paper. **b** Masked 3D classification for the catalytic core. **c** Masked 3D classification for the biogenesis domain. **d** Data processing flowchart for the catalytic core. **e** Data processing flowchart for the catalytic core focusing on the H2A/H2B heterodimer. **f** Data processing flowchart for the biogenesis domain. **g** Data processing flowchart for the biogenesis domain focusing on stems p7-p8a of the hTR RNA.
