# Supplementary material for: Zipper head mechanism of telomere synthesis by human telomerase
Source: Cell Res. 2021 Nov 15;31(12):1275–90. doi: 10.1038/s41422-021-00586-7 (PMC8648750; doi:10.1038/s41422-021-00586-7)
Supplement: Supplementary file 3 — Supplementary information, Figure S3 [file 41422_2021_586_MOESM3_ESM.pdf]

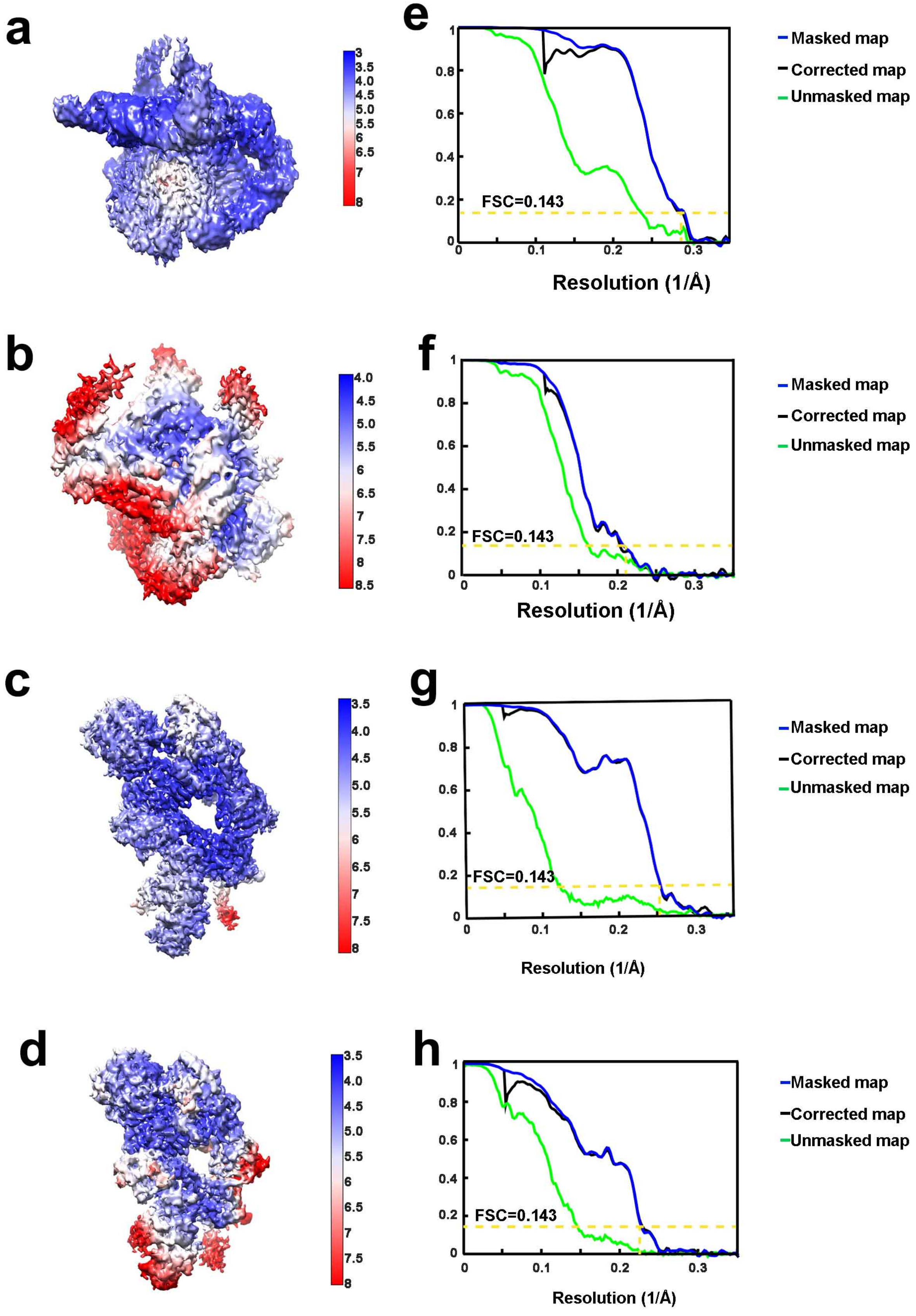
a
3
3.5
4.0
4.5
5.0
5.5
6
6.5
7
8
e
Masked map
Corrected map
Unmasked map
FSC=0.143
Resolution (1/Å)
b
4.0
4.5
5.0
5.5
6
6.5
7
7.5
8
8.5
f
Masked map
Corrected map
Unmasked map
FSC=0.143
Resolution (1/Å)
c
3.5
4.0
4.5
5.0
5.5
6
6.5
7
7.5
8
g
Masked map
Corrected map
Unmasked map
FSC=0.143
Resolution (1/Å)
d
3.5
4.0
4.5
5.0
5.5
6
6.5
7
7.5
8
h
Masked map
Corrected map
Unmasked map
FSC=0.143
Resolution (1/Å)

Supplementary information, Fig. S3 Local resolution estimations of human telomerase holoenzyme. a-d Local resolutions estimated by RELION 3.1 for the four reconstructed maps in Figure S2. **e-f** Gold-standard Fourier Shell Correlation (FSC) curves of the four reconstructed maps, respectively. Resolutions were estimated at FSC = 0.143.
