# Supplementary material for: Zipper head mechanism of telomere synthesis by human telomerase
Source: Cell Res. 2021 Nov 15;31(12):1275–90. doi: 10.1038/s41422-021-00586-7 (PMC8648750; doi:10.1038/s41422-021-00586-7)
Supplement: Supplementary file 4 — Supplementary information, Figure S4 [file 41422_2021_586_MOESM4_ESM.pdf]

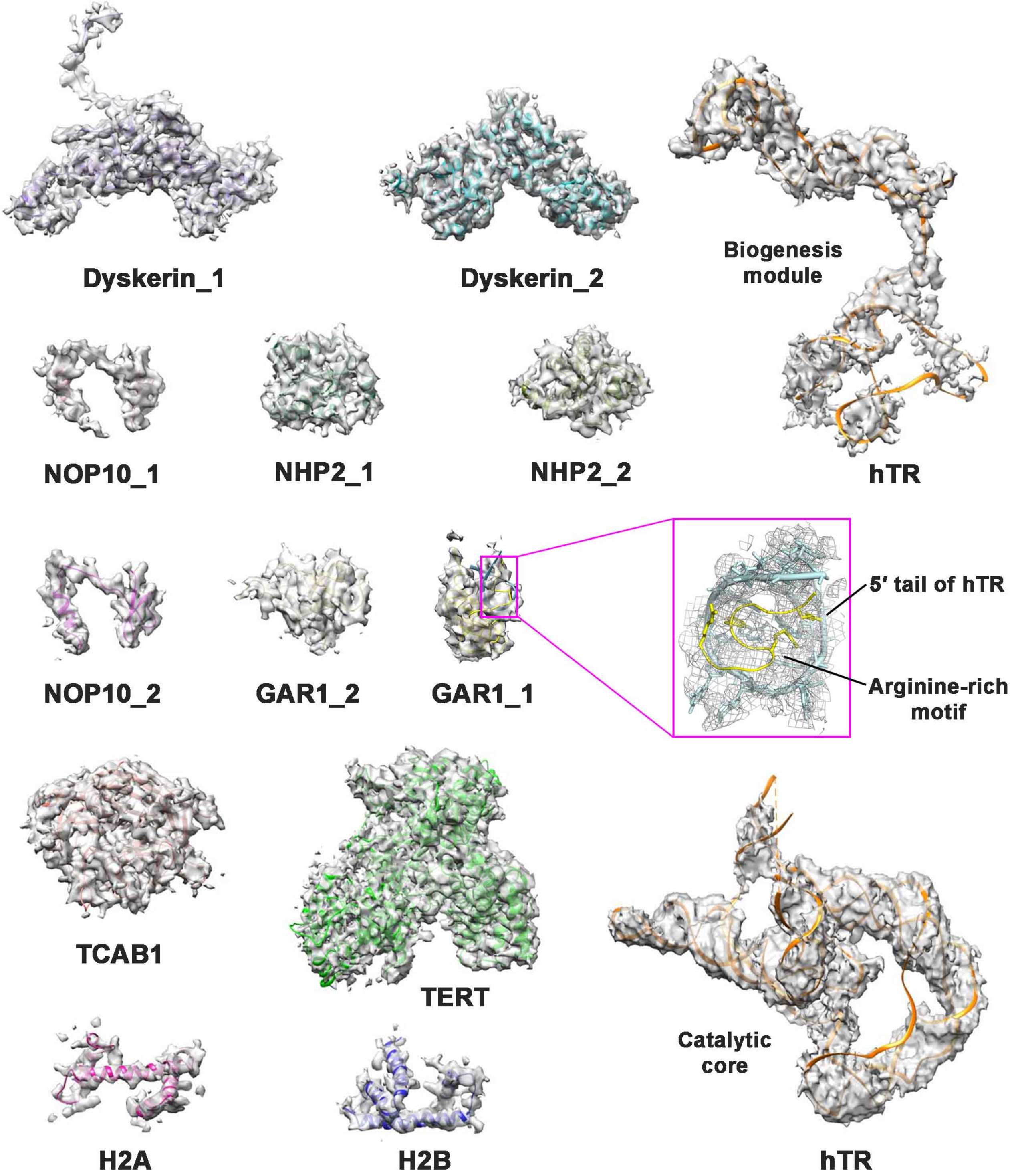
Dyskerin_1
Dyskerin_2
Biogenesis module
NOP10_1
NHP2_1
NHP2_2
hTR
NOP10_2
GAR1_2
GAR1_1
5′ tail of hTR
Arginine-rich motif
TCAB1
TERT
Catalytic core
H2A
H2B
hTR

Supplementary information, Fig. S4 Representative cryo-EM density maps for the RNA and protein components of human telomerase. A gallery of representative cryo-EM density maps for all components of human telomerase.
