# Supplementary material for: Zipper head mechanism of telomere synthesis by human telomerase
Source: Cell Res. 2021 Nov 15;31(12):1275–90. doi: 10.1038/s41422-021-00586-7 (PMC8648750; doi:10.1038/s41422-021-00586-7)
Supplement: Supplementary file 5 — Supplementary information, Figure S5 [file 41422_2021_586_MOESM5_ESM.pdf]

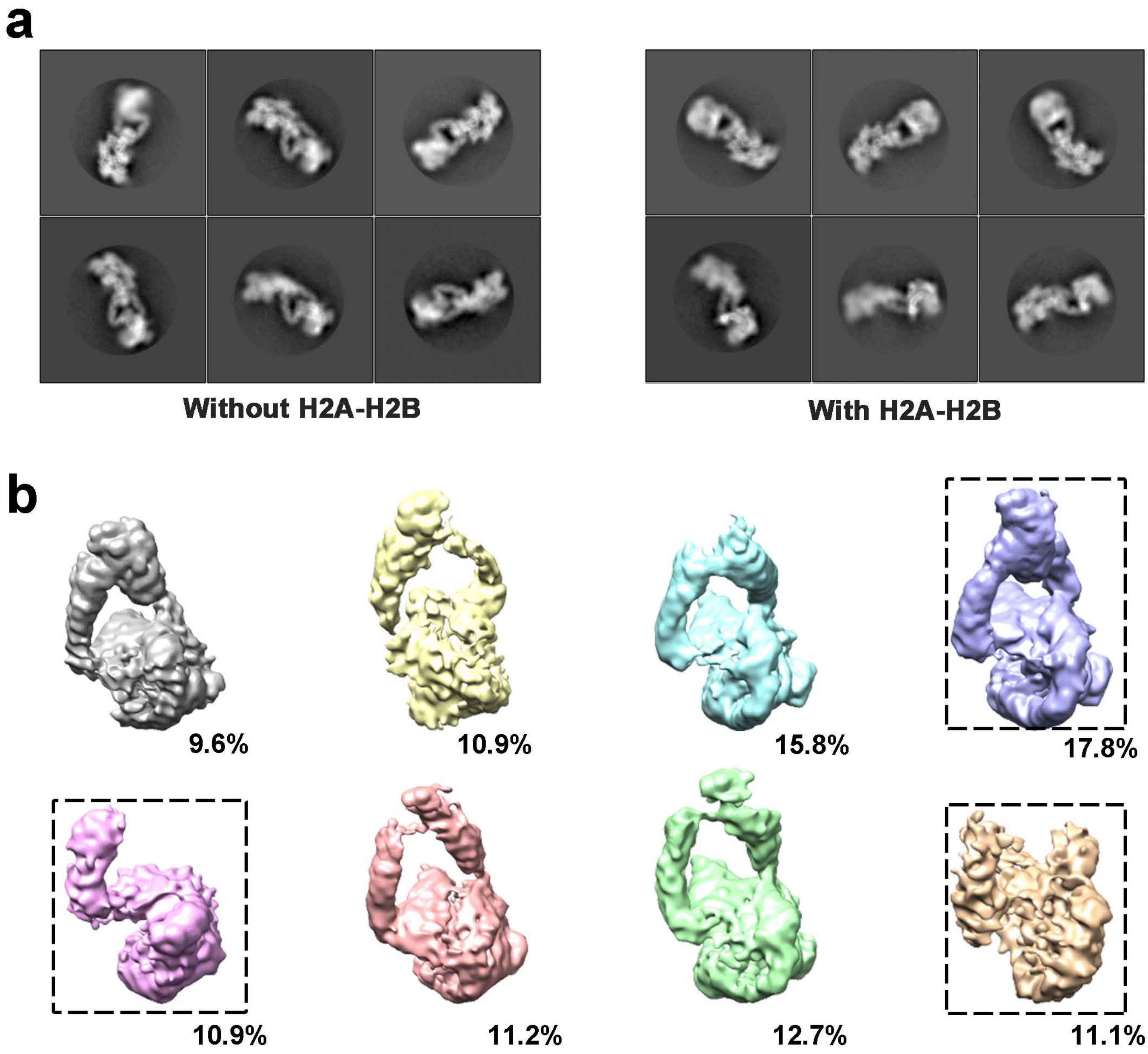
a
Without H2A-H2B
With H2A-H2B
b
9.6%
10.9%
15.8%
17.8%
10.9%
11.2%
12.7%
11.1%

Supplementary information, Fig. S5 2D and 3D image classification of the cryo-EM data processing for the catalytic core. a Selected reference-free 2D class averages of the human telomerase particles with or without the H2A-H2B heterodimer. **b** Masked 3D classification for the catalytic core. Particles without the H2A-H2B heterodimer are boxed with dashed rectangles.
