# Supplementary material for: Zipper head mechanism of telomere synthesis by human telomerase
Source: Cell Res. 2021 Nov 15;31(12):1275–90. doi: 10.1038/s41422-021-00586-7 (PMC8648750; doi:10.1038/s41422-021-00586-7)
Supplement: Supplementary file 6 — Supplementary information, Figure S6 [file 41422_2021_586_MOESM6_ESM.pdf]

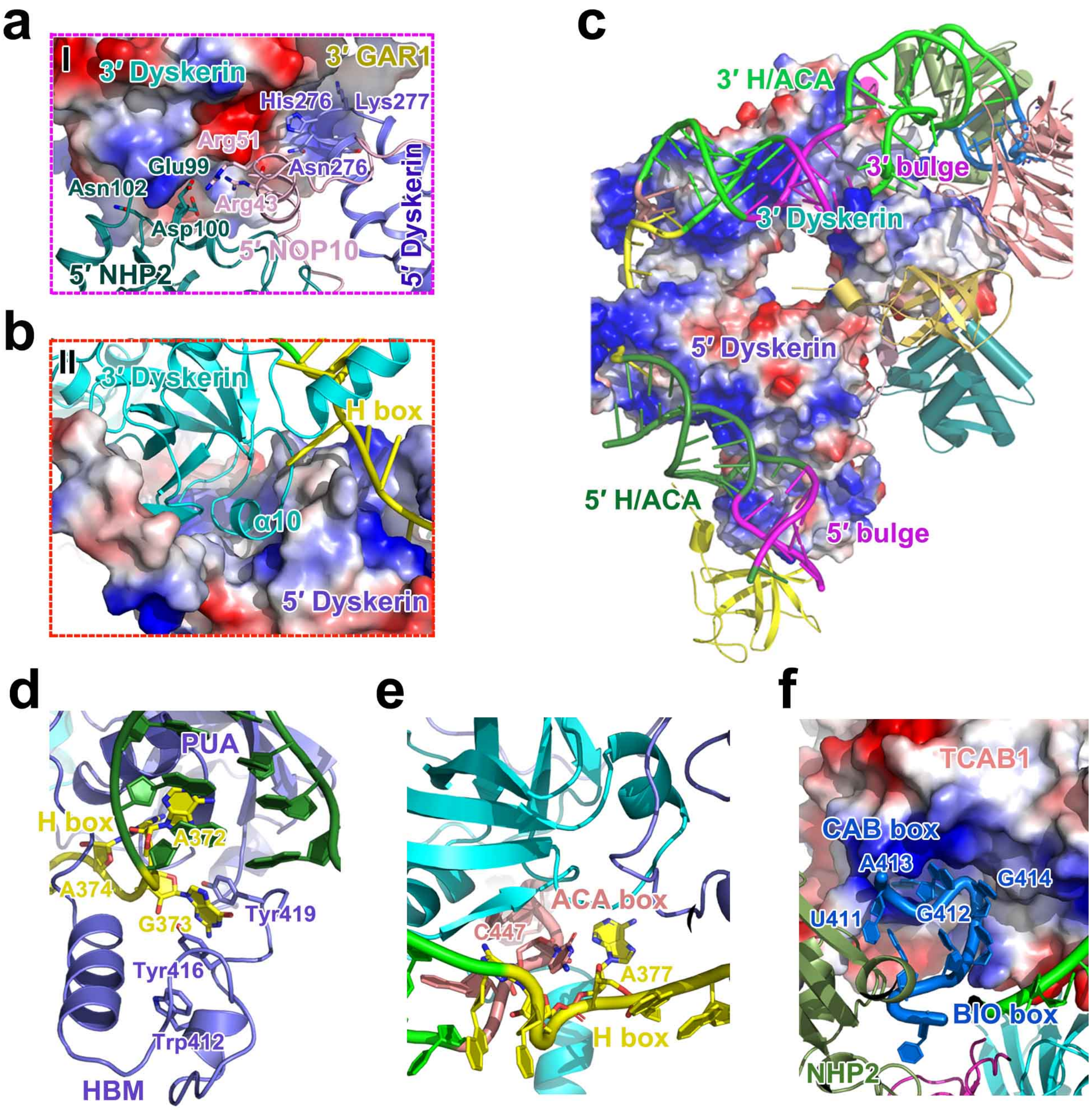
a
I
3′ Dyskerin
3′ GAR1
His276
Lys277
Arg51
Glu99
Asn276
Asn102
Arg43
Asp100
5′ NOP10
5′ NHP2
5′ Dyskerin
b
II
3′ Dyskerin
H box
α10
5′ Dyskerin
c
3′ H/ACA
3′ bulge
3′ Dyskerin
5′ Dyskerin
5′ H/ACA
5′ bulge
d
PUA
H box
A372
A374
G373
Tyr419
Tyr416
Trp412
HBM
e
ACA box
C447
A377
H box
f
TCAB1
CAB box
A413
G414
U411
G412
BIO box
NHP2

Supplementary information, Fig. S6 Details of the protein-protein and protein-RNA interactions in the biogenesis domain. a, b Details of the protein-protein interacting interfaces between the two sets of H/ACA units as in Fig. 2a. At interface I, the 3' and 5' H/ACA unit proteins are shown in electrostatic surface and ribbon representations, respectively. Hydrophilic residues are shown in stick model. At interface II, the 3' and 5' dyskerin proteins are shown in ribbon and electrostatic surface representations, respectively. **c** Electrostatic surface representations of the two dyskerin proteins and their interactions with hTR. The hTR RNA is shown in cartoon model, and the 5' and 3' bulges are colored in magenta. **d-f** Closeup views of the H (**d**), ACA (**e**) and CAB (**f**) boxes that are recognized by 5' and 3' dyskerin and TCAB1, respectively. TCAB1 and NHP2 are shown in electrostatic surface ribbon representations respectively, and hTR is shown in cartoon representation.
