# Supplementary material for: Zipper head mechanism of telomere synthesis by human telomerase
Source: Cell Res. 2021 Nov 15;31(12):1275–90. doi: 10.1038/s41422-021-00586-7 (PMC8648750; doi:10.1038/s41422-021-00586-7)
Supplement: Supplementary file 7 — Supplementary information, Figure S7 [file 41422_2021_586_MOESM7_ESM.pdf]

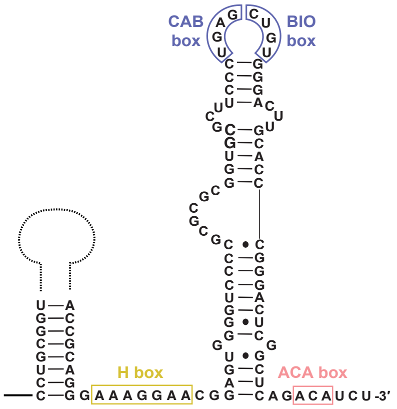

Cattle (*Bos taurus*)

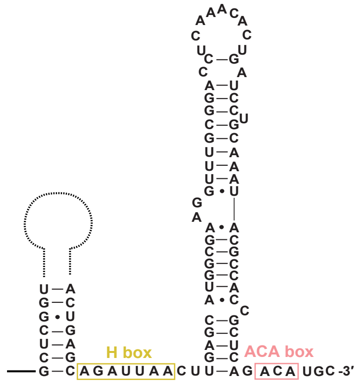

Zebrafish (*Dania rerio*)

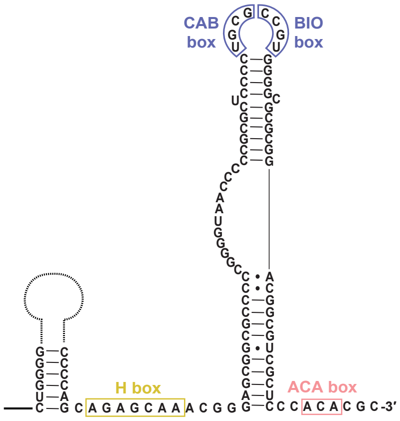

Chicken (*Gallus gallus*)

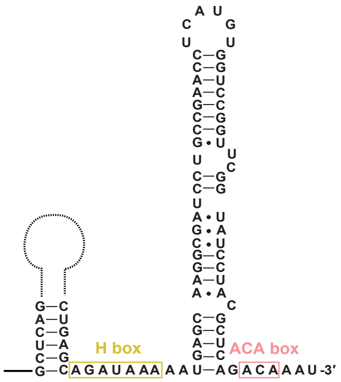

Medaka (*Oryzias latipes*)

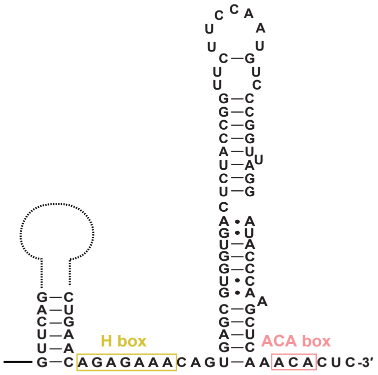

Fugu (*Takifugu rubripes*)

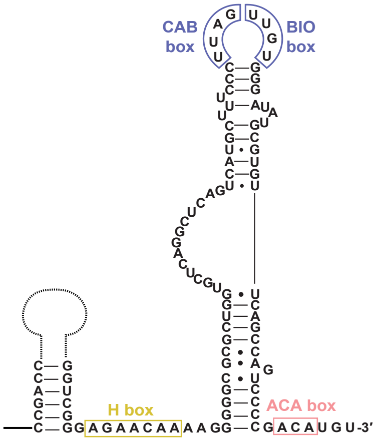

African clawed frog (*Xenopus laevis*)

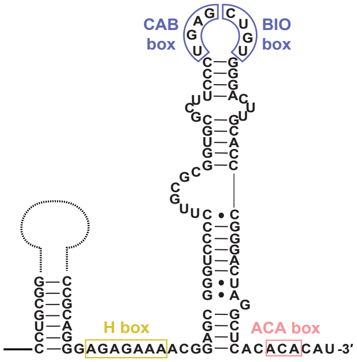

Raccon (*Procyon lotor*)

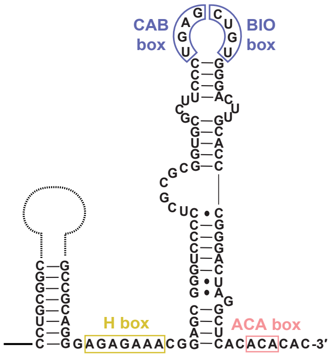

Domestic ferret (*Mustela putorius furo*)

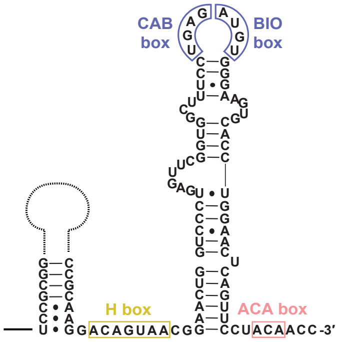

Norway Rat (*Rattus norvegicus*)

Supplementary information, Fig. S7 Schematic diagrams of the RNA secondary structures of the biogenesis domains in vertebrates. The conserved CAB, H and ACA boxes are highlighted in purple, green and blue boxes.
