# Supplementary material for: Zipper head mechanism of telomere synthesis by human telomerase
Source: Cell Res. 2021 Nov 15;31(12):1275–90. doi: 10.1038/s41422-021-00586-7 (PMC8648750; doi:10.1038/s41422-021-00586-7)
Supplement: Supplementary file 8 — Supplementary information, Figure S8 [file 41422_2021_586_MOESM8_ESM.pdf]

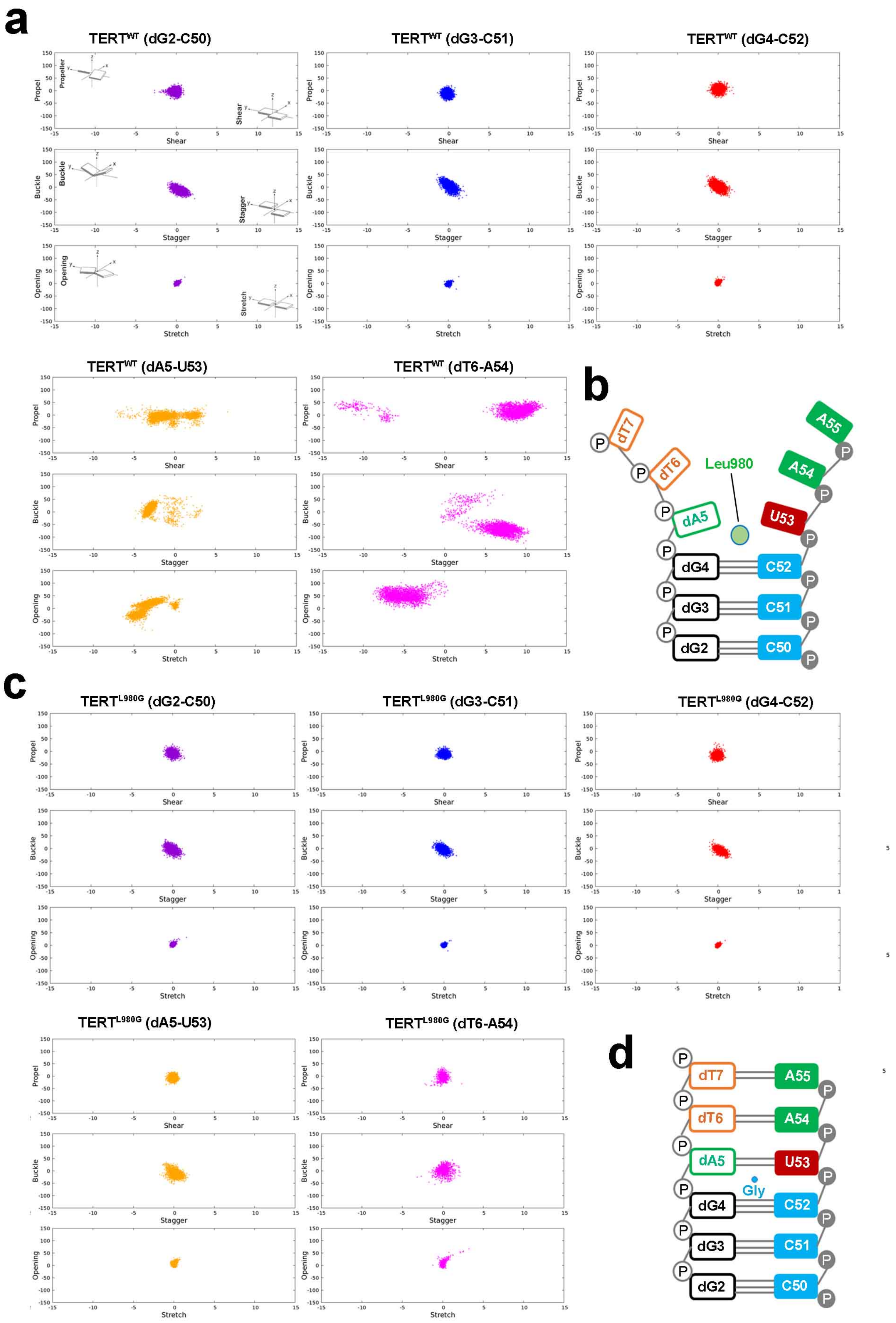
a
TERTWT (dG2-C50)
TERTWT (dG3-C51)
TERTWT (dG4-C52)
Propel
Propeller
Shear
Buckle
Stagger
Opening
Stretch
TERTWT (dA5-U53)
TERTWT (dT6-A54)
b
dT7
dT6
dA5
dG4
dG3
dG2
Leu980
A55
A54
U53
C52
C51
C50
P
c
TERTL980G (dG2-C50)
TERTL980G (dG3-C51)
TERTL980G (dG4-C52)
TERTL980G (dA5-U53)
TERTL980G (dT6-A54)
d
Gly

Supplementary information, Fig. S8 Molecular dynamic simulation of different base pairs of the primer-template duplex. a Scatter plots of the geometrical base pair descriptors of the primer-template duplex at positions 2 to 6 in WT human telomerase. **b** Schematic diagram of the primer-template duplex in WT human telomerase. The zipper head residue Leu980 is designated as a light green circle. **c** Scatter plots of the geometrical base pair descriptors of primer-template duplex at positions 2 to 6 in $TERT^{L980G}$ mutant telomerase. **d** Schematic diagram of the primer-template duplex in $TERT^{L980G}$ mutant telomerase. The glycine residue equivalent to Leu980 is designated as a light blue circle.
