# Supplementary material for: Zipper head mechanism of telomere synthesis by human telomerase
Source: Cell Res. 2021 Nov 15;31(12):1275–90. doi: 10.1038/s41422-021-00586-7 (PMC8648750; doi:10.1038/s41422-021-00586-7)
Supplement: Supplementary file 9 — Supplementary information, Figure S9 [file 41422_2021_586_MOESM9_ESM.pdf]

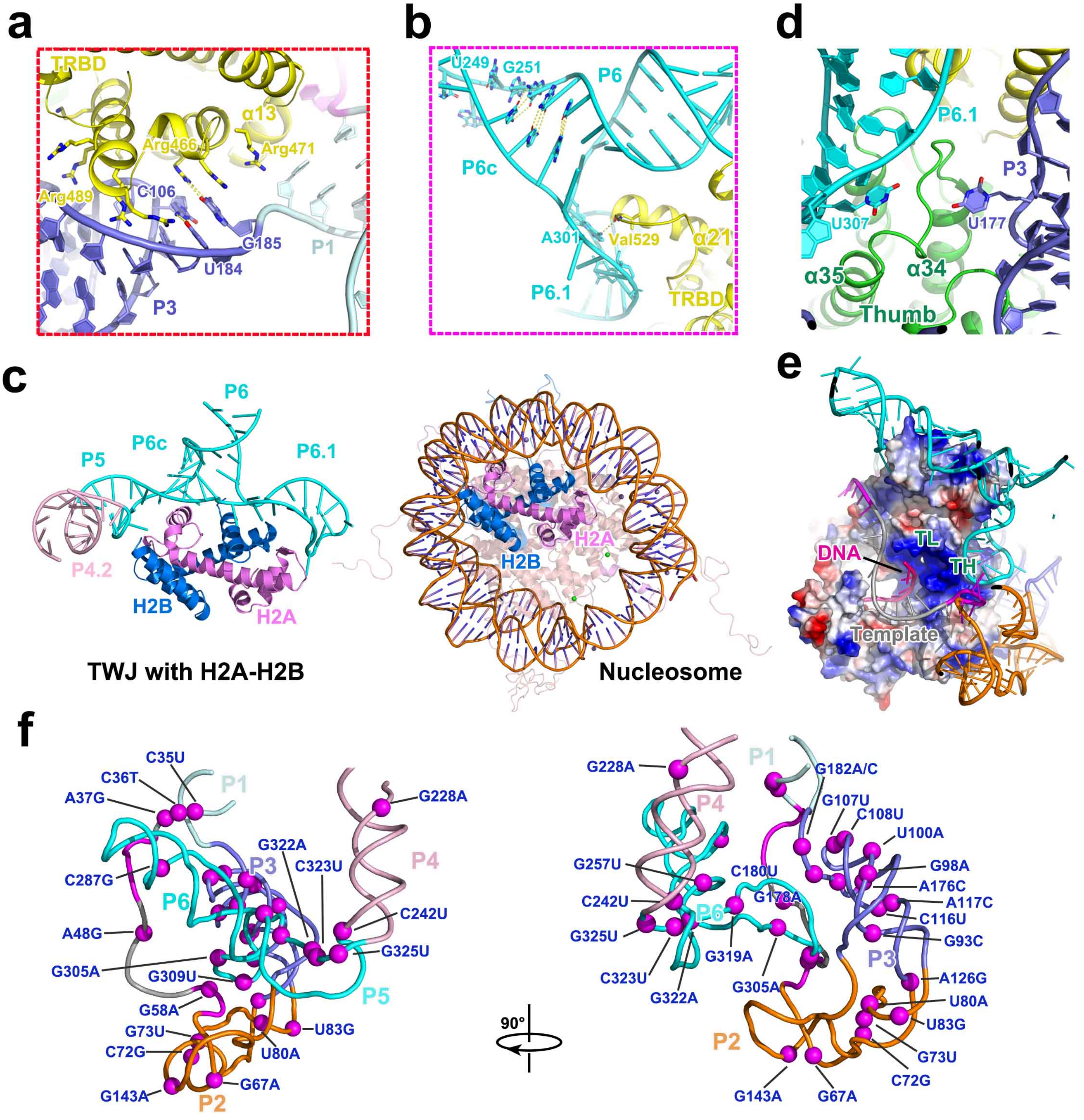
a
TRBD
α13
Arg466
Arg471
Arg489
C106
G185
U184
P1
P3
b
U249
G251
P6
P6c
A301
Val529
α21
P6.1
TRBD
d
P6.1
P3
U307
U177
α35
α34
Thumb
c
P6
P6c
P5
P6.1
P4.2
H2B
H2A
TWJ with H2A-H2B
Nucleosome
e
DNA
TL
TH
Template
f
C35U
C36T
A37G
P1
C287G
P6
P3
G322A
C323U
P4
G228A
C242U
A48G
G325U
G305A
G309U
P5
G58A
G73U
C72G
U80A
U83G
G143A
G67A
P2
90°
G228A
P1
G182A/C
P4
G107U
C108U
U100A
G257U
C180U
G98A
G178A
A176C
C242U
A117C
P6
C116U
G325U
G93C
G319A
C323U
G322A
G305A
P3
A126G
U80A
U83G
P2
G73U
C72G
G143A
G67A

Supplementary information, Fig. S9 Details of the interactions between hTR and TERT. a, b Closeup views of the interactions between the TRBD subdomain of TERT and the t-PK (**a**) and the TWJ (**b**) domains of hTR, respectively. TERT and hTR are shown in ribbon and cartoon representations, respectively. The residues and nucleotides that mediate the interactions are shown in stick model. **c** Structural comparison of H2A/H2B-TWJ and nucleosome structures. **d** Closeup views of the interactions between the thumb subdomain of TERT and the t-PK and TWJ. **e** Electrostatic surface potential of substrate DNA binding site of TERT. Positive potential, blue; negative potential, red. **f** Human disease-derived mutations are mapped onto the corresponding positions in the hTR structure. Single nucleotide substitution mutations are shown in megenta sphere.
