# Supplementary material for: Zipper head mechanism of telomere synthesis by human telomerase
Source: Cell Res. 2021 Nov 15;31(12):1275–90. doi: 10.1038/s41422-021-00586-7 (PMC8648750; doi:10.1038/s41422-021-00586-7)
Supplement: Supplementary file 10 — Supplementary information, Figure S10 [file 41422_2021_586_MOESM10_ESM.pdf]

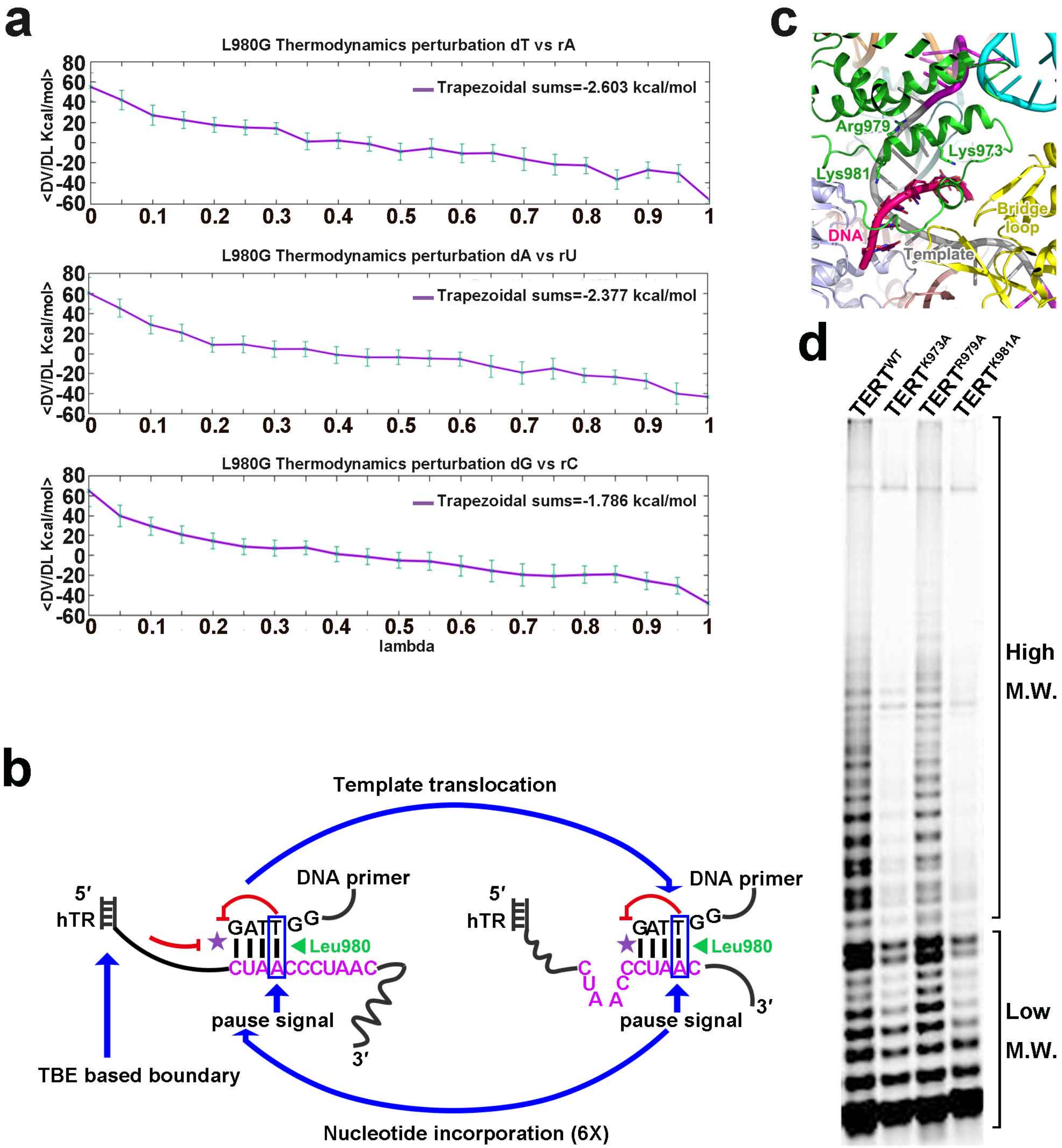
a
L980G Thermodynamics perturbation dT vs rA
Trapezoidal sums=-2.603 kcal/mol
L980G Thermodynamics perturbation dA vs rU
Trapezoidal sums=-2.377 kcal/mol
L980G Thermodynamics perturbation dG vs rC
Trapezoidal sums=-1.786 kcal/mol
<DV/DL Kcal/mol>
lambda
b
Template translocation
DNA primer
5′
hTR
GATT
GG
Leu980
CUAACCCUAAC
pause signal
3′
TBE based boundary
Nucleotide incorporation (6X)
CCUAAC
C U A A C
c
Arg979
Lys973
Lys981
DNA
Template
Bridge loop
d
TERT^WT
TERT^K973A
TERT^R979A
TERT^K981A
High M.W.
Low M.W.

Supplementary information, Fig. S10 Biochemical and Computational analysis of zipper head-based template boundary signal. a Comparison of $\langle dV/d\lambda \rangle_{\lambda}$ values for the alchemical transformation of L980G facing with dT-A, dA-U and dG-C base pairs at position 5, respectively. **b** Coincidence of the zipper-head-based template boundary (blue box) with the physical constrain of TBE stem P1b. Human telomerase uses the rA:dT base pair (blue box) as a pausing signal after completion of each cycle of telomeric repeat addition, referred to as a sequence-based template boundary signal. During a full extension cycle of a telomere repeat, the zipper head Leu980 would experience an unfavorable dG-to-dT transition at position 5, suggestive of an important role of Leu980 in determining the sequence-based template boundary signal. The zipper-head-based template boundary coordinates with TBE-defined boundary to ensure template translocation at the end of each telomere extension cycle to facilitate RAP. The catalytic center and Leu980 are denoted with purple star and green triangle, respectively. **c** Closeup view of the thumb helix and bridge loop in the catalytic cavity. **d** Telomerase activity of purified human telomerase reconstituted from U2OS cells over-expressing Flag-tagged WT and mutant TERT proteins.
