# Supplementary material for: Zipper head mechanism of telomere synthesis by human telomerase
Source: Cell Res. 2021 Nov 15;31(12):1275–90. doi: 10.1038/s41422-021-00586-7 (PMC8648750; doi:10.1038/s41422-021-00586-7)
Supplement: Supplementary file 11 — Supplementary information, Figure S11 [file 41422_2021_586_MOESM11_ESM.pdf]

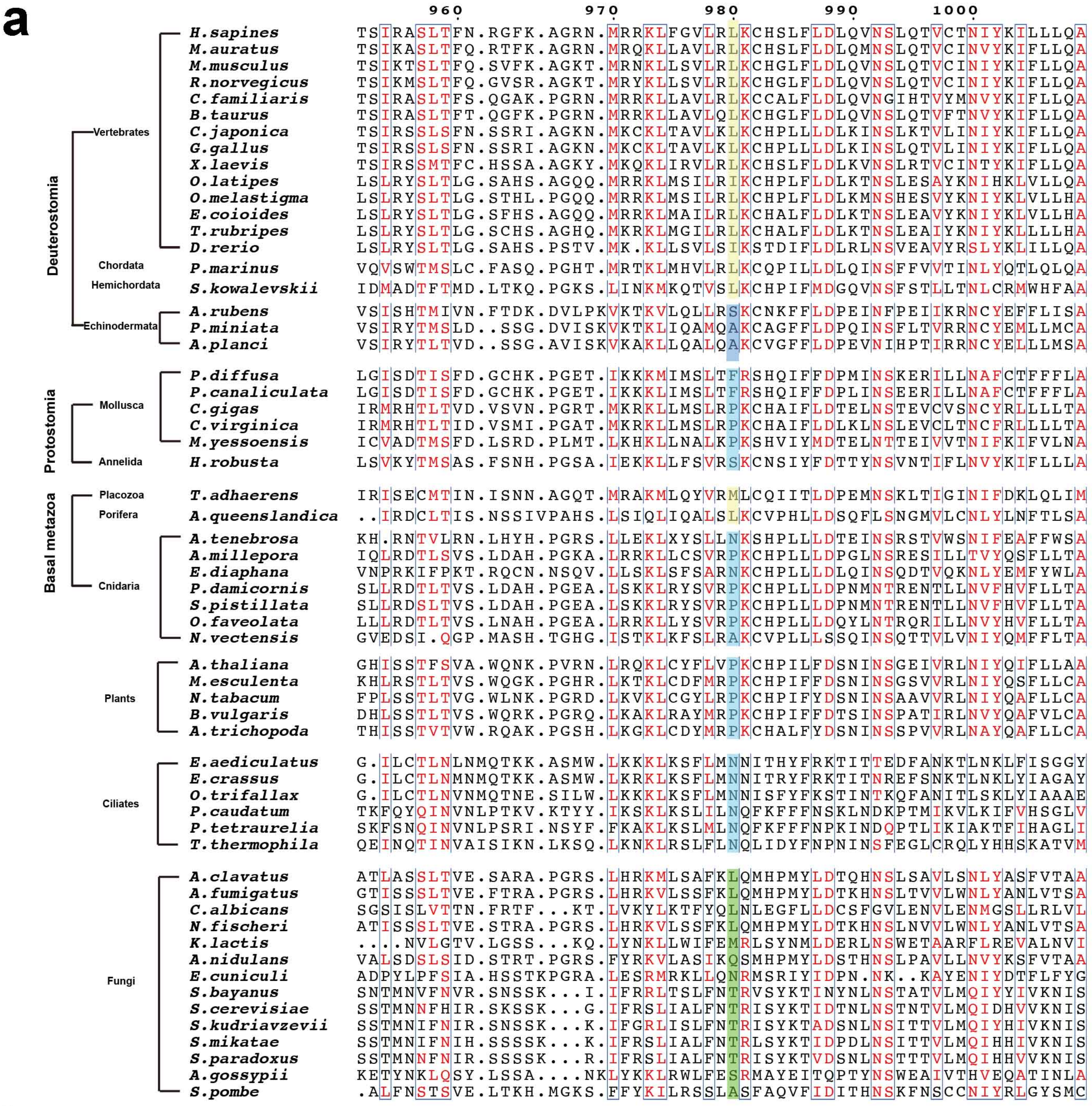
a
960
970
980
990
1000
Deuterostomia
Vertebrates
Chordata
Hemichordata
Echinodermata
Protostomia
Mollusca
Annelida
Basal metazoa
Placozoa
Porifera
Cnidaria
Plants
Ciliates
Fungi
H.sapines TSIRASLTFN.RGFK.AGRN.MRRKLFGVLRLKCHSLFLDLQVNSLQTVCTNIYKILLLQA
M.auratus TSIKASLTFQ.RTFK.AGRN.MRQKLLAVLRLKCHSLFLDLQMNSLQTVCINVYKIFLLQA
M.musculus TSIKTSLTFQ.SVFK.AGKT.MRNKLLSVLRLKCHGLFLDLQVNSLQTVCINIYKIFLLQA
R.norvegicus TSIKMSLTFQ.GVSR.AGKT.MRYKLLSVLRLKCHGLFLDLQVNSLQTVCINIYKIFLLQA
C.familiaris TSIRASLTFS.QGAK.PGRN.MRRKLLAVLRLKCCALFLDLQVNGIHTVYMNVYKIFLLQA
B.taurus TSIRASLTFT.QGFK.PGRN.MRRKLLAVLQLKCHGLFLDLQVNSLQTVFTNVYKIFLLQA
C.japonica TSIRSSLSFN.SSRI.AGKN.MKCKLTAVLKLKCHPLLLDLKINSLKTVLINIYKIFLLQA
G.gallus TSIRSSLSFN.SSRI.AGKN.MKCKLTAVLKLKCHPLLLDLKINSLQTVLINIYKIFLLQA
X.laevis TSIRSSMTFC.HSSA.AGKY.MKQKLIRVLRLKCHSLFLDLKVNSLRTVCINTYKIFLLQA
O.latipes LSLRYSLTLG.SAHS.AGQQ.MRRKLMSILRIKCHPLFLDLKTNSLESAYKNIHKLVLLQA
O.melastigma LSLRYSLTLG.STHL.PGQQ.MRRKLMSILRLKCHPLFLDLKMNSHESVYKNIYKLVLLHA
E.coioides LSLRYSLTLG.SFHS.AGQQ.MRRKLMAILRLKCHALFLDLKTNSLEAVYKNIYKLVLLHA
T.rubripes LSLRYSLTLG.SCHS.AGHQ.MKRKLMGILRLKCHALFLDLKTNSLEAIYKNIYKLLLLHA
D.rerio LSLRYSLTLG.SAHS.PSTV.MK.KLLSVLSIKSTDIFLDLRLNSVEAVYRSLYKLLILLQA
P.marinus VQVSWTMSLC.FASQ.PGHT.MRTKLMHVLRLKCQPILLDLQINSFFVVTINLYQTLQLQA
S.kowalevskii IDMADTFTMD.LTKQ.PGKS.LINKMKQTVSLKCHPIFMDGQVNSFSTLLTNLCRMWHFAA
A.rubens VSISHTMIVN.FTDK.DVLPKVKTKVLQLLRSKCNKFFLDPEINFPEIIKRNCYEFFLISA
P.miniata VSIRYTMSLD..SSG.DVISKVKTKLIQAMQAKCAGFFLDPQINSFLTVRRNCYEMLLMCA
A.planci VSIRYTLTVD..SSG.AVISKVKAKLLQALQAKCVGFFLDPEVNIHPTIRRNCYELLLMSA
P.diffusa LGISDTISFD.GCHK.PGET.IKKKMIMSLTFRSHQIFFDPMINSKERILLNAFCTFFLA
P.canaliculata LGISDTISFD.GCHK.PGET.IKKKLIMSLTFRSHQIFFDPLINSEERILLNAFCTFFLA
C.gigas IRMRHTLTVD.VSVN.PGRT.MKRKLLMSLRPKCHAIFLDTELNSTEVCVSNCYRLLLLTA
C.virginica IRMRHTLTID.VSMI.PGAT.MKRKLLMSLRPKCHAIFLDLKLNSLEVCLTNCFRLLLLTA
M.yessoensis ICVADTMSFD.LSRD.PLMT.LKHKLLNALKPKSHVIYMDTELNTTEIVVTNIFKIFVLNA
H.robusta LSVKYTMSAS.FSNH.PGSA.IEKKLLFSVRSKCNSIYFDTTYNSVNTIFLNVYKIFLLLA
T.adhaerens IRISECMTIN.ISNN.AGQT.MRAKMLQYVRMLCQIITLDPEMNSKLTIGINIFDKLQLIM
A.queenslandica ..IRDCLTIS.NSSIVPAHS.LSIQLIQALSLKCVPHLLDSQFLSNGMVLCNLYLNFTLSA
A.tenebrosa KH.RNTVLRN.LHYH.PGRS.LLEKLXYSLLNKSHPLLLDTEINSRSTVWSNIFEAFFWSA
A.millepora IQLRDTLSVS.LDAH.PGKA.LRRKLLCSVRPKCHPLLLDPGLNSRESILLTVYQSFLLTA
E.diaphana VNPRKIFPKT.RQCN.NSQV.LLSKLSFSARNKCHPLLLDLQINSQDTVQKNLYEMFYWLA
P.damicornis SLLRDTLTVS.LDAH.PGEA.LSKKLRYSVRPKCHPLLLDPNMNTRENTLLNVFHVFLLTA
S.pistillata SLLRDSLTVS.LDAH.PGEA.LSKKLRYSVRPKCHPLLLDPNMNTRENTLLNVFHVFLLTA
O.faveolata LLLRDTLTVS.LNAH.PGEA.LRRKLLYSVRPKCHPLLLDQYLNTRQRILLNVYHVFLLTA
N.vectensis GVEDSI.QGP.MASH.TGHG.ISTKLKFSLRAKCVPLLLSSQINSQTTVLVNIYQMFFLTA
A.thaliana GHISSTFSVA.WQNK.PVRN.LRQKLCYFLVPKCHPILFDSNINSGEIVRLNIYQIFLLAA
M.esculenta KHLRSTLTVS.WQGK.PGHR.LKTKLCDFMRPKCHPIFFDSNINSGSVVRLNIYQSFLLCA
N.tabacum FPLSSTLTVG.WLNK.PGRD.LKVKLCGYLRPKCHPIFYDSNINSAAVVRLNIYQAFLLCA
B.vulgaris DHLSSTLTVS.WQRK.PGRQ.LKAKLRAYMRPKCHPIFFDTSINSPATIRLNVYQAFVLCA
A.trichopoda THISSTVTVW.RQAK.PGSH.LKGKLCDYMRPKCHALFYDSNINSSPVVRLNAYQAFLLCA
E.aediculatus G.ILCTLNLNMQTKK.ASMW.LKKKLKSFLMNNITHYFRKTITTEDFANKTLNKLFISGGY
E.crassus G.ILCTLNMNMQTKK.ASMW.LKRKLKSFLMNNITRYFRKTITNREFSNKTLNKLYIAGAY
O.trifallax G.ILCTLNVNMQTNE.SILW.LKKKLKSFLMNNISFYFKSTINTKQFANITLSKLYIAAAE
P.caudatum TKFQYQINVNLPTKV.KTYY.IKSKLKSLILNQFKKFFNSKLNDKPTMIKVLKIFVHSGLV
P.tetraurelia SKFSNQINVNLPSRI.NSYF.FKAKLKSLMLNQFKKFFNPKINDQPTLIKIAKTFIHAGLI
T.thermophila QEINQTINVAISIKN.LKSQ.LKNKLRSLFLNQLIDYFNPNINSFEGLCRQLYHHSKATVM
A.clavatus ATLASSLTVE.SARA.PGRS.LHRKMLSAFKLQMHPMYLDTQHNSLSAVLSNLYASFVTAA
A.fumigatus GTISSSLTVE.FTRA.PGRS.LHRKVLSSFKLQMHPMYLDTKHNSLTVVLWNLYANLVTSA
C.albicans SGSISLVTTN.FRTF...KT.LVKYLKTFYQLNLEGFLLDCSFGVLENVLENMGSLLRLVL
N.fischeri ATISSSLTVE.STRA.PGRS.LHRKVLSSFKLQMHPMYLDTKHNSLNVVLWNLYANLVTSA
K.lactis ....NVLGTV.LGSS...KQ.LYNKLLWIFEMRLSYNMLDERLNSWETAARFLREVALNVI
A.nidulans VALSDSLSID.STRT.PGRS.FYRKVLASIKQSMHPMYLDSTHNSLPAVLLNVYKSFVTAA
E.cuniculi ADPYLPFSIA.HSSTKPGRA.LESRMRKLLQNRMSRIYIDPN.NK..KAYENIYDTFLFYG
S.bayanus SNTMNVFNVR.SNSSK...I.IFRRLTSLFNTRVSYKTINYLNSSTATVLMQIYYIVKNIS
S.cerevisiae SSTMNNFHIR.SKSSK...G.IFRSLIALFNTRISYKTIDTNLNSTNTVLMQIDHVVKNIS
S.kudriavzevii SSTMNIFNIR.SNSSK...K.IFGRLISLFNTRISYKTADSNLNSITTVLMQIYHIVKNIS
S.mikatae SSTMNIFNIH.SSSSK...K.IFRSLIALFNTRISYKTIDPDLNSITTVLMQIHHIVKNIS
S.paradoxus SSTMNNFNIR.SSSSK...R.IFRSLIALFNTRISYKTVDSNLNSTTTVLMQIHHVVKNIS
A.gossypii KETYNKLQSY.LSSA....NKLYKKLRWLFESRMAYEITQPTYNSWEAIVTHVEQATINLA
S.pombe .ALFNSTSVE.LTKH.MGKS.FFYKILRSSLASFAQVFIDITHNSKFNSCCNIYRLGYSMC

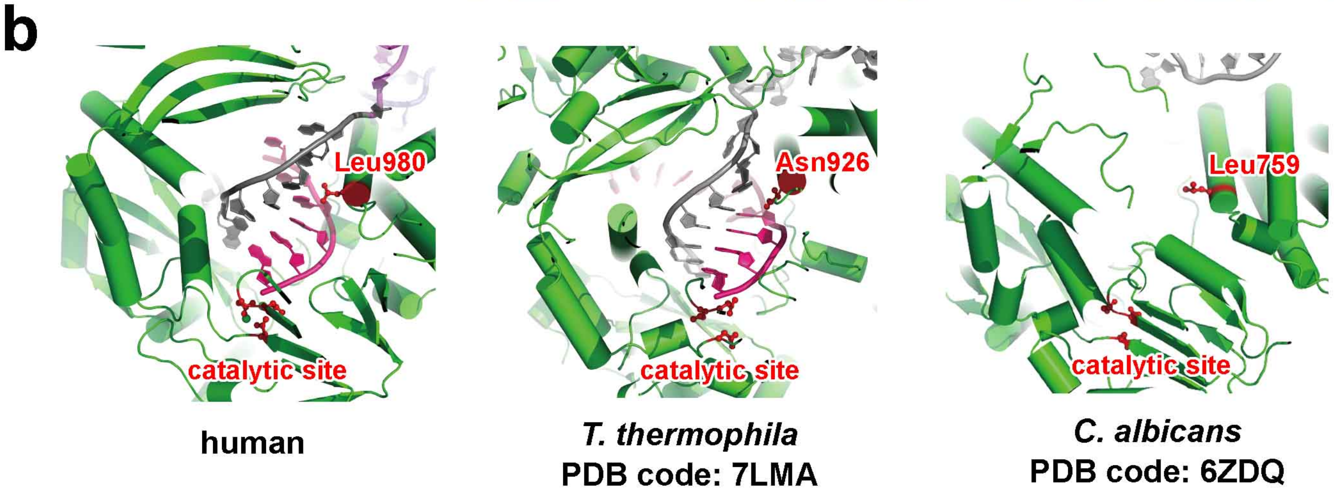
b
Leu980
catalytic site
Asn926
catalytic site
Leu759
catalytic site
human
T. thermophila
PDB code: 7LMA
C. albicans
PDB code: 6ZDQ

Supplementary information, Fig. S11 Conservation of zipper-head mechanism in eukaryotes. a Multiple sequence alignment of the thumb-helix-flanking sequence of TERT proteins from various eukaryotes. Positions equivalent to human $TERT^{Leu980}$ are highlighted with respective colors. **b** Structural comparison analysis of the active sites in human, *Tetrahymena thermophila* and *Candida albicans* TERT proteins. The three essential aspartate residues in the catalytic centers and the zipper head residues (Leu980 in human TERT, Asn926 in *T. thermophila* TERT and Leu766 in *C. albicans* TERT) are denoted.
