# Supplementary material for: Zipper head mechanism of telomere synthesis by human telomerase
Source: Cell Res. 2021 Nov 15;31(12):1275–90. doi: 10.1038/s41422-021-00586-7 (PMC8648750; doi:10.1038/s41422-021-00586-7)
Supplement: Supplementary file 12 — Supplementary information, Table S1 [file 41422_2021_586_MOESM12_ESM.pdf]

Supplementary information, Table S1

Cryo-EM data collection, refinement and validation statistics.

	Core<br>EMD-31811<br>PDB 7V99	Histone_masked<br>EMD-31812	HACA<br>EMD-31813<br>PDB 7V9A	GAR1_masked<br>EMD-31814
**Data collection and processing**				
Magnification	81,000	81,000	81,000	81,000
Voltage (kV)	300	300	300	300
Electron exposure (e–/Å^2)	62	62	62	62
Defocus range (μm)	-1.5 to -2.8	-1.5 to -2.8	-1.5 to -2.8	-1.5 to -2.8
Pixel size (Å)	1.1	1.1	1.1	1.1
Symmetry imposed	C1	C1	C1	C1
Initial particle images (no.)	771,617	771,617	771,617	771,617
Final particle images (no.)	297,526	50,600	167,923	131,717
Map resolution (Å)	3.54	4.97	3.94	4.40
FSC threshold	0.143	0.143	0.143	0.143
Map resolution range (Å)	2.8-4.4	3.0-5.0	3.5-7.5	4.4-7.5
**Refinement**				
Initial model used (PDB code)	6D6V		3HAY	
Model resolution (Å)	4.80		4.99	
FSC threshold	0.143		n/a	
Model resolution range (Å)	n/a		n/a	
Map sharpening *B* factor (Å^2)	-57.1		-40.3	
Model composition				
Non-hydrogen atoms	14,582		16,876	
Protein residues	1,170		1,723	
Nucleotide	249		146	
B factors (Å^2)				
Protein	59.8		49.7	
Nucleotide	79.2		65.7	
R.m.s. deviations				
Bond lengths (Å)	0.002		0.002	
Bond angles (°)	0.515		0.567	
Validation				
MolProbity score	1.83		1.74	
Clashscore	7.85		6.87	
Poor rotamers (%)	0.10		0.00	
Ramachandran plot				
Favored (%)	94.06		94.78	
Allowed (%)	5.85		5.22	
Disallowed (%)	0.09		0.00	
