# Supplementary material for: Zipper head mechanism of telomere synthesis by human telomerase
Source: Cell Res. 2021 Nov 15;31(12):1275–90. doi: 10.1038/s41422-021-00586-7 (PMC8648750; doi:10.1038/s41422-021-00586-7)
Supplement: Supplementary file 14 — Supplementary Video Note [file 41422_2021_586_MOESM14_ESM.pdf]

Supplementary information, Movie S1 Coupled seesaw-like conformational changes of fingers A and B. The conformational changes of fingers A and B were derived from principle component analysis (PCA). Proteins and nucleic acids are shown in cylinder and cartoon representations, respectively. Fingers-A and fingers-B are colored in salmon and deep teal, respectively.
